# Monitoring egg fertility, embryonic morbidity, and mortality in an oviparous elasmobranch using ultrasonography

**DOI:** 10.3389/fvets.2024.1410377

**Published:** 2024-07-30

**Authors:** Lance Adams, Jennifer T. Wyffels, Brittney Goodwin, Rachel Munson, Louise LeBorgne, Kevin A. Feldheim, Kady Lyons

**Affiliations:** ^1^Aquarium of the Pacific, Long Beach, CA, United States; ^2^Ripley’s Aquariums, Orlando, FL, United States; ^3^Delaware Biotechnology Institute, Center for Bioinformatics and Computational Biology, University of Delaware, Newark, DE, United States; ^4^Pritzker Laboratory for Molecular Systematics and Evolution, The Field Museum, Chicago, IL, United States; ^5^Center for Species Survival, Georgia Aquarium, Atlanta, GA, United States

**Keywords:** embryonic development, growth, abnormality, parthenogenesis, ultrasound

## Abstract

Ultrasonography is widely used to monitor pregnancy in viviparous species, but it is underutilized as a tool to characterize embryonic development in oviparous species. Currently, a multi-institutional effort is underway to re-wild the endangered zebra shark (*Stegostoma tigrinum*) to locations where this species was previously extirpated by leveraging the reproductive efforts of aquarium sharks as a source of brood stock. Zebra sharks are oviparous and fecund, but a large percentage of their yolked eggs do not result in hatchlings. Therefore, ultrasonography represents a potential tool for distinguishing fertile eggs with developing embryos from degrading eggs, and to diagnose changes in early embryonic development predictive of poor outcomes. The objectives of the current study were to use ultrasonography to assess egg fertility, monitor early embryonic development, and identify morphological indicators that may be predictive of early embryonic mortality. Freshly laid eggs from four female zebra sharks were collected and inventoried daily at Aquarium of the Pacific. Eggs were incubated undisturbed for 2 to 4 weeks and subsequently examined weekly via ultrasound to assess fertility and monitor embryo development. Among 120 fertile eggs, embryos were identified as early as 8 days post-oviposition, with average (±SD) time to first observation at 30 ± 7 days. Morphological and behavioral abnormalities were observed for most embryos (*n* = 84, 70%) as early as 16 days and up to 95 days post-oviposition. Common abnormalities included: bent or curled tails, vesicle(s) at the base of the yolk stalk, and slow or weak movement. Only one embryo survived to hatch during the study and was genetically-confirmed parthenogenetic, suggesting hatching success for parthenotes is low (<1%). Ultrasonography was demonstrated to be an effective and non-invasive method to determine egg fertility, identify embryos with developmental abnormalities, and monitor embryo growth.

## 1 Introduction

Characterization of reproductive cycling and associated life history parameters is important for management of both domesticated and non-domesticated animals (1, 2). Non-invasive ultrasonography has been a crucial tool in a variety of reproductive applications. For example, gonadal changes can be monitored to determine peak periods of reproductive activity in both males and females (3, 4), information that can then inform livestock production (5, 6) or characterize natural breeding cycles of wild mammals (7–9) and reptiles (10) in human care. Ultrasound has also been used to estimate fecundity and to determine sex of teleost fish (11) as well as monitor reproductive maturity, reproductive cycles and pregnancy or egg laying of both viviparous (12–15) and oviparous (16, 17) elasmobranchs, negating the need for lethal sampling which is especially advantageous for species of conservation concern.

Monitoring embryonic development through non-lethal techniques is another aspect where ultrasonography has advanced the field of reproductive science, both in humans and animals. In little-studied species, ultrasonography has been used to establish developmental baselines (18, 19), particularly for species where lethal sampling is not possible or advisable. For example, in elephants (*Loxodonta africana* and *Elephas maximus*) ultrasound was used to monitor early embryonic development from conception to implantation and determined that these species have delayed implantation (20). Understanding developmental timelines during early elephant development allowed the authors to more accurately stage embryonic development described in previous studies where gestational time was unknown. For species with well-described embryonic developmental timelines (e.g., humans, livestock, etc), ultrasound monitoring is important for confirming if (or when) embryos reach significant landmarks at the appropriate time during their gestation (21, 22). As such, ultrasonography has been used as a diagnostic tool to detect and predict signs of congenital problems during embryo development. For example, in humans, ultrasonography has been used to detect prenatal cleft deformities (23) and improper development of the central nervous system (24). In livestock, ultrasonography was used to monitor for embryonic malformations induced as a result of dams ingesting poisonous plants, information that alerted ranchers of potential hazards on farm lands (25).

As most applications have occurred in viviparous species, ultrasonography as a tool to characterize embryonic development in oviparous species is underutilized. In the chicken (*Gallus gallus*), ultrasonography allowed real-time monitoring of heart formation in developing chicks, representing a non-lethal way to study organogenesis in this model species (26, 27). Also in chickens, ultrasonography has enabled biomedical researchers using the chorioallantoic-membrane assay to track *in ovo* tumor growth and size over time (28). Nevertheless, the use of ultrasonography to track aspects of embryonic development in other oviparous species has been limited.

Among elasmobranch fishes (sharks, skates, and rays), approximately 40% of species are oviparous and their embryonic development has traditionally been monitored in two ways. Embryos can be identified and monitored through a process called “candling,” where a bright backlight is shown through the egg case. Candling is used to confirm fertility in early incubation and check for viability throughout incubation, but elucidating detailed information on embryo morphology (beyond gross morphometrics) is limited for most species by the opacity of their egg shell/case. Similar techniques are used to monitor avian and reptilian embryonic development (29, 30). Changes in embryo morphology can also be tracked by “windowing” the egg case (i.e., cutting an opening) to expose the embryo for viewing. In elasmobranchs, windowing is less risky to the embryo after it has gone through eclosion, the process whereby the egg case respiratory fissures naturally open and seawater circulates through the egg case which occurs after approximately 40% of their total incubation (31). However, by this point in incubation a significant portion of development is complete, excluding most embryonic stages from direct observation. Windowing prior to eclosion often results in mortality due to iatrogenic infections, even when egg cases are resealed with a transparent barrier (Wyffels, pers. obs).

Other, more advanced, techniques have also been applied to study embryo morphogenesis *in ovo*, but present their own limitations. For example, magnetic resonance imaging (MRI) was recently used to characterize early development in chickens (embryonic day 1–20) longitudinally (32). Likewise, micro-computed tomography (micro-CT) has been applied to study chick cardiac organogenesis (33). While both of these methods are promising, equipment access, expertise, and monetary constraints may limit the use of these techniques in non-model species. Ultrasonography can overcome some of these limitations and allows a non-destructive internal view at any time during development but is especially advantageous for early development. Use of ultrasonography to examine embryo development has been applied to two Heterodontus species, which lay rather thick egg cases that render candling rather ineffective (34). Therefore, ultrasonography fills a significant gap in non-lethal longitudinal monitoring techniques for the earliest stages of embryonic growth and development in oviparous elasmobranch species.

Ultrasonography may also help fill outstanding needs in the conservation community where methods to more easily access fertility in species of concern are required (18, 35). Within the field of elasmobranch conservation, a multi-institutional effort is underway to re-wild the endangered zebra shark (*Stegostoma tigrinum*) to locations where this species was previously extirpated by leveraging aquarium collections as a source of brood stock (36). Through this workplan, eggs from genetically appropriate adults will be shipped to Indonesia where developing embryos will be hatched and reared prior to release. However, while zebra sharks are fecund (37), a large percentage of yolked eggs degrade within the first few weeks post-oviposition (38). Therefore, ultrasonography represents a potential tool that could be used to accurately distinguish fertile from non-fertile eggs, monitor early embryonic development, and diagnose changes in the egg and embryo predictive of poor outcomes. Because the resources that can be invested in each individual egg for potential re-wilding are limited, developing a diagnostic “early warning system” via ultrasound will help guide efforts to achieve the goals of this conservation initiative. The objectives of this study were to use ultrasonography to (1) assess egg fertility, (2) monitor early embryonic development and progress after fertility is confirmed, and (3) identify developmental indicators that may be predictive of early embryonic mortality.

## 2 Methods

### 2.1 Husbandry

Four adult female zebra sharks were maintained at Aquarium of the Pacific where egg laying activities were monitored. Two females were housed in an approximately 1.4 million liter mixed-tropical fish indoor exhibit with artificial lighting (12 h light:12 h dark) supplemented by natural light via skylights. The other two females were housed separately in an outdoor ~0.41 million liter exhibit exposed to natural light. All exhibits were filled with natural, filtered seawater kept at 23.8–25°C. Diet consisted of thawed seafood including clam, squid, mackerel and herring fed at ~7% percent of body weight weekly, along with a multivitamin supplement tablet (Mazuri Vita-Zu Shark/Ray; formula 5 M24). Throughout the study, a concurrent, but separate, effort was underway to conduct artificial insemination trials with these females.

### 2.2 Egg collection and incubation

Exhibits were monitored daily, and freshly laid eggs were removed by aquarists during routine cleaning. Egg cases were labeled with sequentially numbered tags attached through the non-hatching end of the egg case (Supplementary File 1), and the date of oviposition was recorded along with purported female, which was determined based on collection exhibit, egg shape and routine physical examinations to identify females that were actively laying. Eggs were relocated to an independent system maintained with continuously-moving seawater (23.8–25°C). Eggs were incubated horizontally and, on a platform, elevated off the bottom to allow water to circulate around the entire case.

### 2.3 Ultrasound monitoring

Eggs were incubated undisturbed for up to 4 weeks (~28–32 days) and afterwards underwent weekly checks to monitor for fertility and embryo development. Pre-ecolosion eggs were briefly exposed to air during transfer from the incubation system to a 4 L acrylic rectangular container (Kritter Keeper, Lees Aquarium & Pet Products, San Marcos, CA) with seawater, while eggs that had undergone eclosion were removed from the incubation system completely submersed to prevent air intrusion. Eggs were maintained in the same general orientation as incubation throughout the procedure to minimize movement of the animal pole, making it easier to locate embryos on the upper surface of the yolk via ultrasound. Eggs were examined using a Sonosite Edge II ultrasound and a 15–6 MHz linear transducer (HFL50, Fuifilm Sonosite Inc., Bothell, WA) with general resolution and small parts settings selected. The transducer was submerged in the water which served as the coupling agent between the transducer and the egg. Each egg was imaged in both transverse and sagittal views moving from the non-hatching end to the hatching end or from either lateral keel (lateral margin) towards the other until the entire content of the egg case was imaged (Supplementary File 2).

At each exam, eggs were evaluated for yolk integrity, presence/absence of an embryo, and echogenicity of perivitelline fluid (anechoic or particulate; Supplementary File 3). If the yolk was intact and no embryo was observed, eggs were allowed to incubate further. Eggs that fouled (i.e., formation of a slimy film on the external egg case with particulate perivitelline fluid) prior to confirmation of an embryo were discarded. Once eggs were confirmed fertile, embryos were measured weekly using the ultrasound’s caliper tool for total length (cm) and the appearance of developmental features (e.g., external gills, heartbeat, etc.) was noted. The first appearance of morphological abnormalities was documented and subsequently monitored at each exam. Embryo movement was categorized as steady, slow, or none (Supplementary File 4). Non-motile embryos were allowed to incubate 1 week further to recheck and confirm mortality. After weekly exams, eggs were returned to their incubation enclosures.

### 2.4 Genetic testing

Embryo mortality was assessed through egg fouling, loss of embryo motility or heart beat, or observed disintegration of the yolk. When possible, necropsies were performed to sample embryonic tissue for genetic testing to determine parentage. DNA was extracted from samples using a salting-out method employed in other studies (39, 40). Embryos were genotyped using 14 previously published microsatellite markers (41). In addition, two new microsatellite loci (Sfa325 and Sfa371) were developed from the enriched library of Dudgeon et al. (41) (Supplementary Table 1). PCRs of the 14 published loci were carried out as previously described. For Sfa325 and Sfa371, PCRs were performed in 10 μl volumes with 1x PCR buffer (10 mM Tris–HCl, 50 mM KCl, pH 8.3), 0.12 mM of each dNTP, 10x BSA, 1.5 mM MgCl_2_, 1 U Taq polymerase, 0.04 μM forward primer tagged with an M13 sequence on the 5′ end (42), 0.16 μM of both the species-specific reverse primer, and a fluorescently labeled M13 primer. Thermal cycling proceeded as follows: an initial denaturation step of 94°C for 4 min was followed by 30 cycles of 94°C for 15 s, 58°C for 15 s, and 72°C for 45 s, followed by 8 cycles of 94°C for 15 s, 53°C for 15 s, and 72°C for 45 s. A final elongation step of 72°C for 10 min concluded each PCR. All PCR products were run with an internal ladder [ALEXA-725, (43)] and on an ABI 3730XL DNA Analyzer (Thermo Fisher Scientific, Waltham, Massachusetts). Individuals were genotyped using Geneious v.10.0.3^1^ (44).

### 2.5 Data analysis

Descriptive statistics (mean ± standard deviation) were used to characterize early life history parameters including: time to first embryo observance (days between oviposition and first identification of an embryo), time to first identification of developmental landmarks or other morphological characteristics, and lifespan (days between oviposition and embryo expiration). Embryo length was measured from still images and growth monitored by taking the mean length of embryos at the same incubation time point grouped by week. Embryos were evaluated for the presence/absence and frequency of morphological abnormalities, and lifespan was compared between groups (i.e., those with an abnormality versus those without) using a Wilcoxon U-test.

## 3 Results

Weekly ultrasonography of incubating eggs commenced in November 2021 and concluded in December 2023. During that time, the embryonic development of 120 embryonated eggs was examined longitudinally using ultrasonography. Embryos were visually identifiable on the surface of the yolk with viability confirmed by their sinusoidal movements, which began early in development (Supplementary File 5). Initially, a subset of eggs (*n* = 13) was examined weekly after oviposition to determine the earliest time point an embryo could be detected. For this subset of eggs, embryos were observed as early as 8 days post-oviposition, with mean time to first observation occurring at 18 ± 3 days (~2.5 weeks). However, most eggs (*n* = 107, 89%) were incubated for 3 to 5 weeks before their first ultrasound examination. With all eggs included, embryos were observed by 30 (±6) days post-oviposition (~4 weeks). The longest incubation time before an embryo was observed was 47 days post-oviposition.

During examinations, the appearance of the perivitelline fluid surrounding the developing embryo was noted as either anechoic fluid or heterogeneous fluid containing particulates (Supplementary File 6). On ultrasound, presence of particulate material gives the visual appearance of a “snow globe.” Particulate material was observed in 50 cases (42% of all eggs), with first observation ranging from 26 to 74 days post-oviposition (median = 41 days).

### 3.1 Growth and developmental landmarks

**Figure 1:**
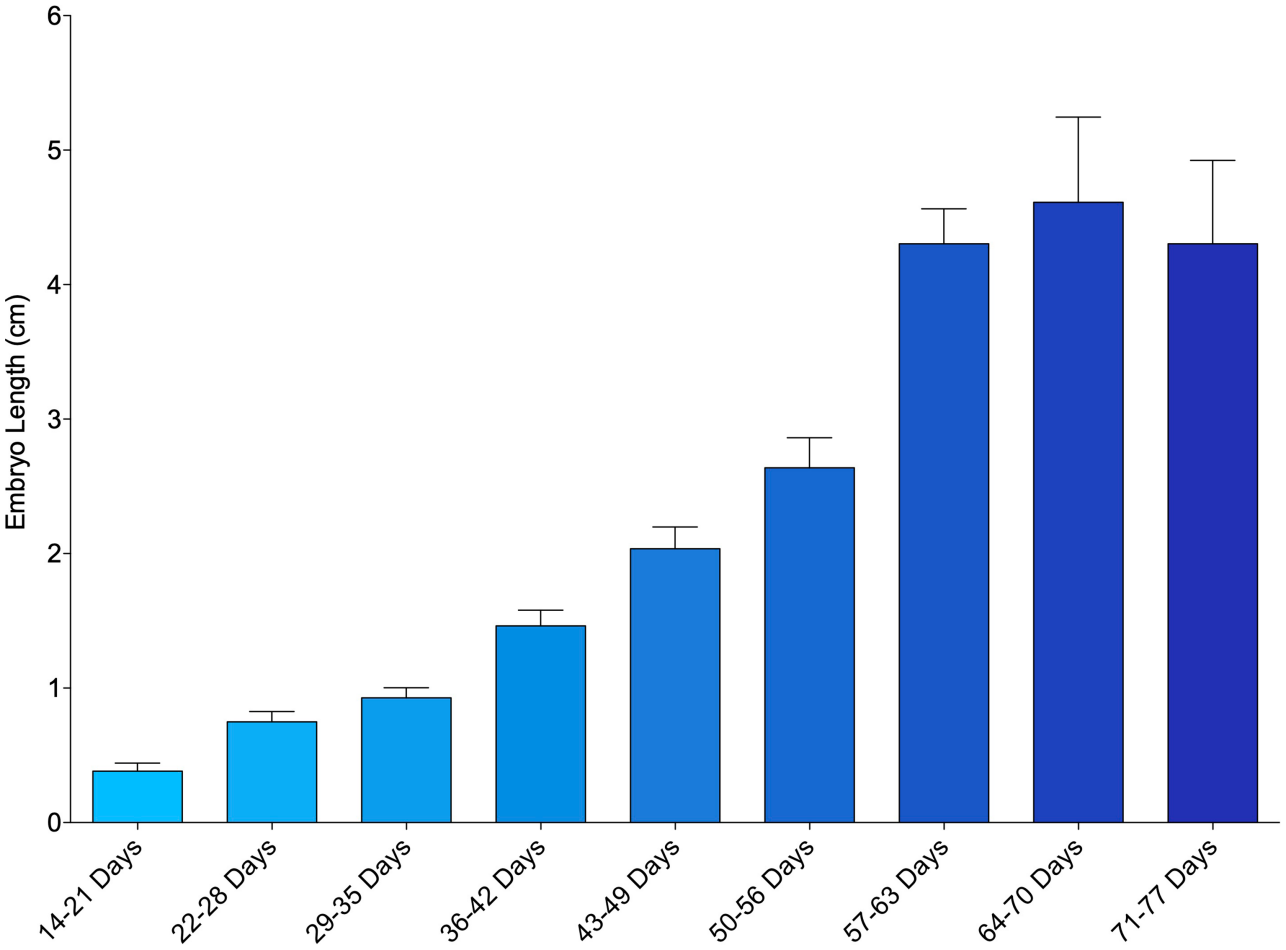
Mean length of zebra shark (*Stegostoma tigrinum*) embryos as measured via ultrasound using the caliper tool. Embryos were grouped by week of development.

The smallest measured embryo was 0.18 cm and observed from an egg that incubated 23 days before its first examination. Mean embryo length generally increased over the course of the study from week to week (Figure 1; Supplementary File 7). In a small number of instances (27 out of 224 measurements taken, 12%), embryo length decreased between successive time points and these inconsistencies tended to occur earlier in development. Embryo length was measurable from first detection to about 64–70 days post-oviposition (~9 weeks), after which obtaining an accurate measurement became too difficult due to their size and positioning within the egg case (Figure 1).

**Figure 2:**
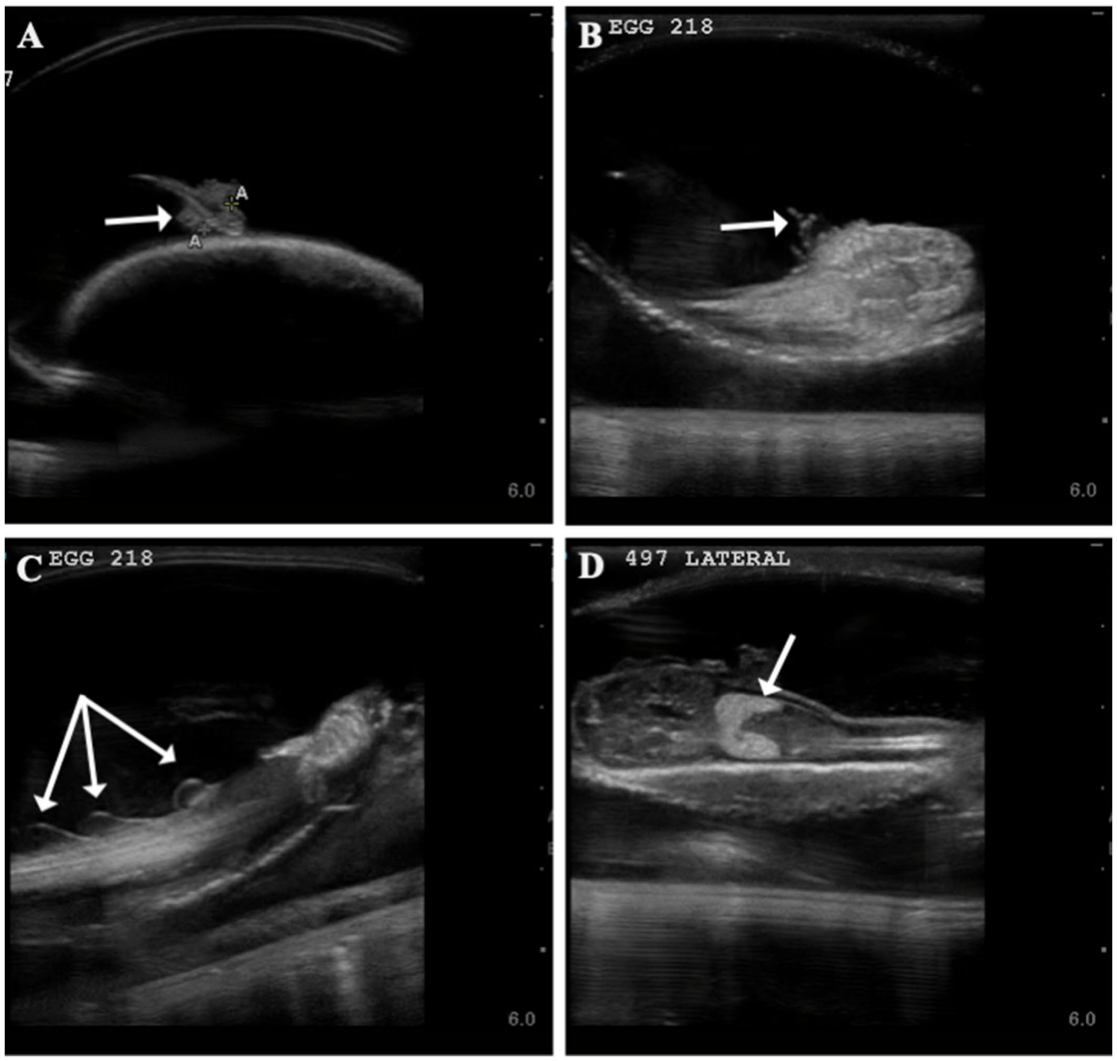
Developmental landmarks (denoted with white arrow) identified on ultrasound for two zebra shark (*Stegostoma tigrinum*) embryos (Egg # 218 and 497) at two different time points. Change in external gills from 60 days post-oviposition **(A)** to 93 days post-oviposition **(B)**, note that embryos featured are two different individuals. Fin folds **(C)** seen on an embryo 100 days post-oviposition and liver **(D)** identified on an embryo 116 days post-oviposition.

Time to first observance of easily recognized developmental features via ultrasonography were recorded. External gill filaments were observed 66 ± 19 (*n* = 14) days post-oviposition (9.5 weeks; Figure 2). A heartbeat was detectable in two embryos at (or between) 61 and 114 days post-oviposition and the mouth could be seen between 103 and 114 days (Supplementary Table 2). As early as 177 days post-oviposition, the embryo was large enough that its movement was restricted so that the pelvic fins could be visualized in an attempt to determine sex. As claspers were not clearly identifiable, the embryo was presumed to be female and later confirmed female at hatching; however, no male embryos were available for comparison in this study. In the latter half of development, post-eclosion, the liver was observable and heart and buccal pump rate could be quantified via ultrasound (Figure 2; Supplementary File 8).

### 3.2 Embryo abnormalities

**Figure 3:**
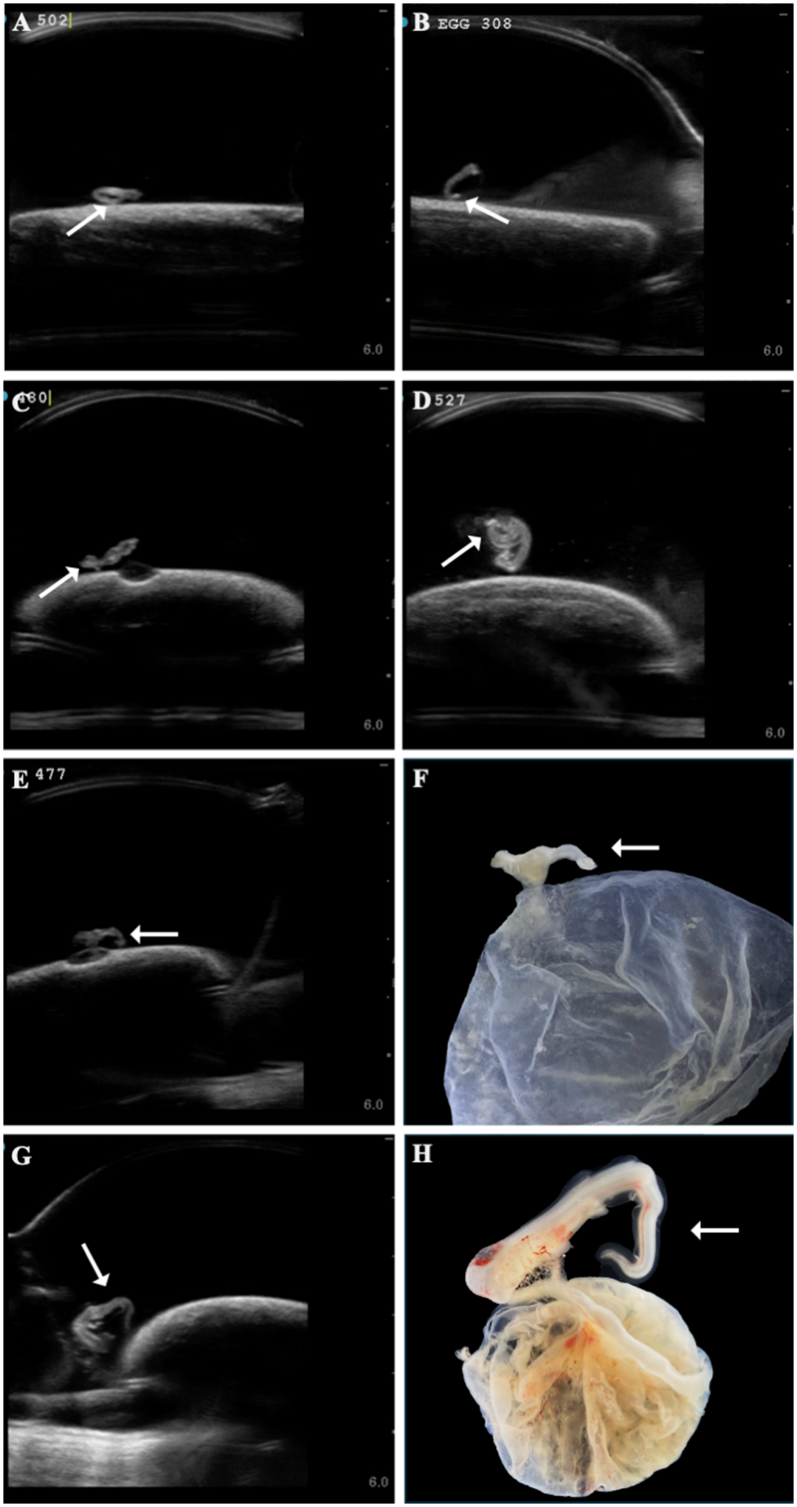
A range of different zebra shark (*Stegostoma tigrinum*) embryo abnormal tail morphologies (white arrows) were observed over the course of the study on ultrasound at 37, 47, 64, and 79 days post-oviposition, respectively **(A–D)**. In a subset of eggs, tail abnormalities were confirmed on dissection (**E,F**, 45 days post-oviposition; **G,H**, 69 days post-oviposition).

**Figure 4:**
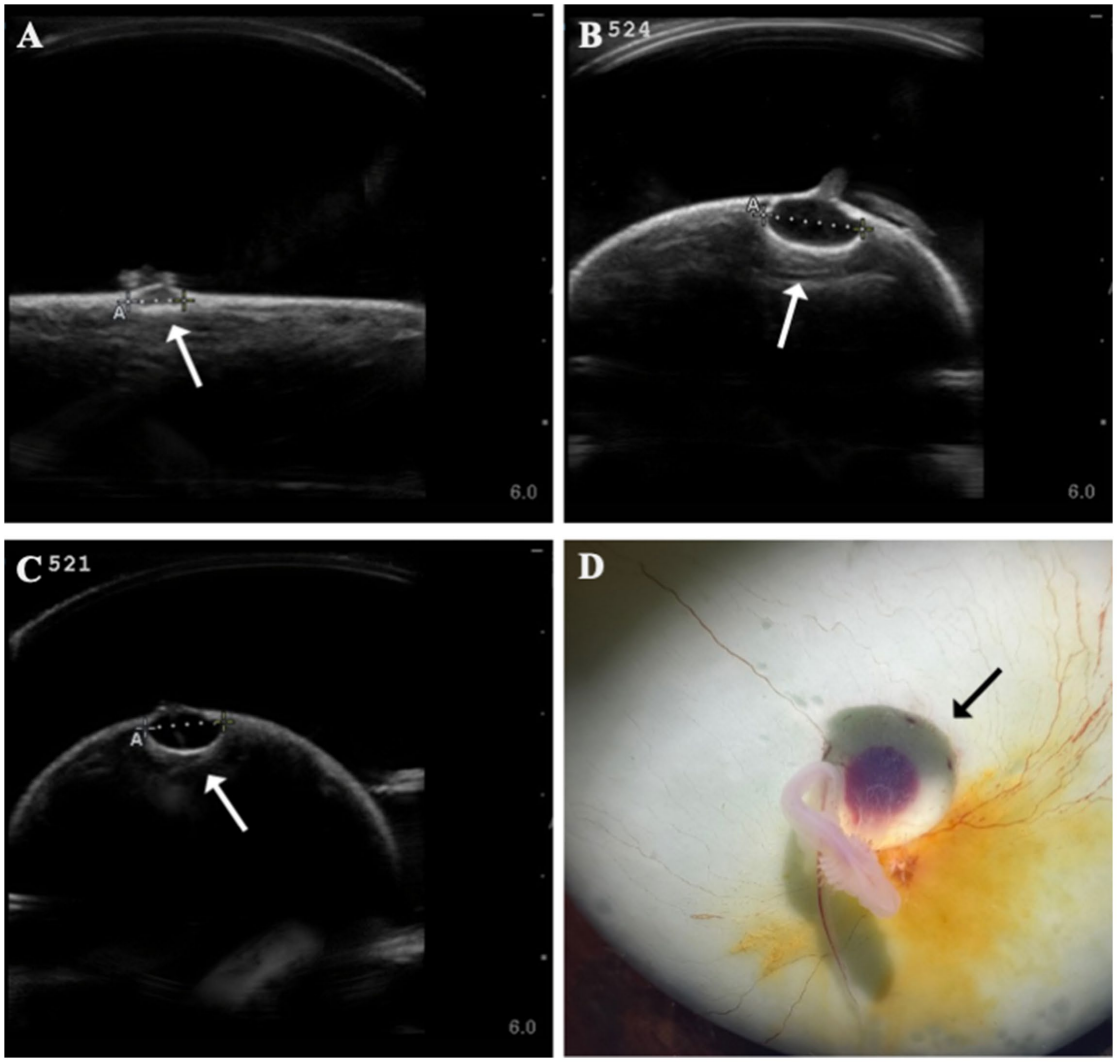
Formation of anechoic fluid-filled vesicles (arrows) at the base of the yolk stalk as seen on ultrasound in zebra shark (*Stegostoma tigrinum*) eggs **(A–C)**. The vesicle was documented to expand in the same egg (#524) from 44 days post-oviposition **(A)** to 79 days post-oviposition **(B)**. Vesicles were confirmed at necropsy **(D)**.

**Figure 5:**
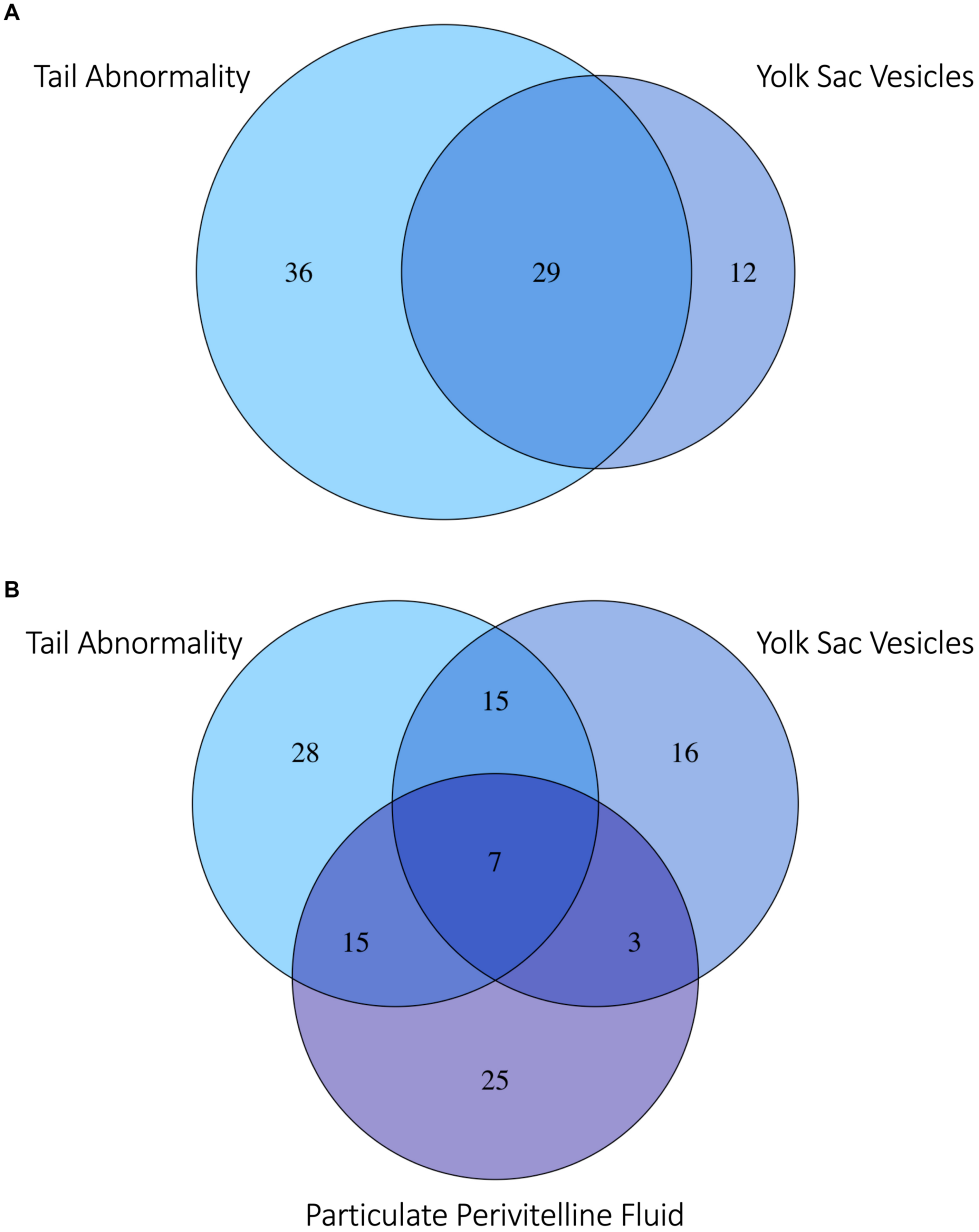
Venn diagram of **(A)** zebra shark (*Stegostoma tigrinum*) embryos with the top two abnormalities observed: Abnormal tail deformities tails (blue) and anechoic yolk sac vesicles (violet). **(B)** Embryos with the top two most frequently observed abnormalities and/or flocculent perivitelline fluid (purple) observed during development. Numbers in each bubble or overlap region are numbers of embryos with one or more abnormalities observed.

Several developmental anomalies were commonly observed including embryos with a sharp bend or coil in the tail, anechoic fluid pockets or vesicles on the yolk sac at the base of the yolk stalk, and more rarely those with a disproportionate or oddly shaped cranium and/or underdeveloped gill filaments. When possible, abnormalities were physically confirmed at the time of egg dissection. Out of 120 embryonated eggs, 84 embryos (70%) had a deformity detectable using ultrasonography. In general, abnormalities were detected early in development (43 ± 15 days post-oviposition, ~6 weeks). The most frequently observed deformity (*n* = 65, 54%) was a bend or coil in the tail (Figure 3; Supplementary File 9), followed by a vesicle at the base of the umbilicus (*n* = 41; Figure 4). The least common abnormality observed was related to head shape and proportion (*n* = 6). Multiple deformities were observed in approximately 50% of embryos (Figure 5A), and often they were detected within 2 to 3 weeks of the first observance of the embryo (bent/coiled tail = 13 ± 12 days; vesicles = 18 ± 14 days; oddly-shaped cranium = 20 ± 9 days). When a developmental anomaly was observed, it did not resolve with time. For example, once a yolk sac vesicle was detected on ultrasound, the diameter of it either increased or stayed the same size on subsequent exams (Supplementary Files 10, 11).

### 3.3 Embryo morbidity and mortality

**Figure 6:**
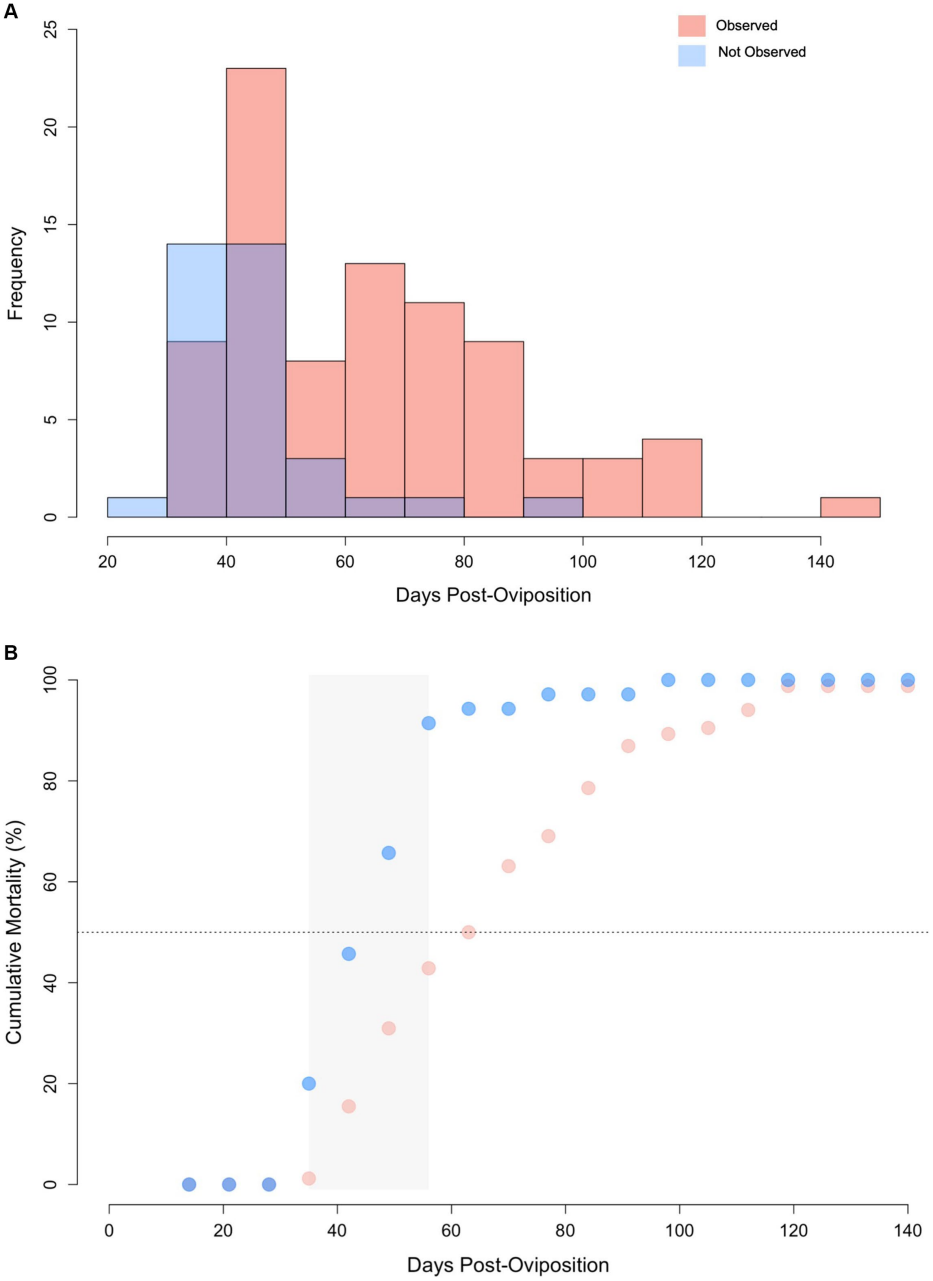
**(A)** Histogram of lifespan for zebra shark (*Stegostoma tigrinum*) embryos with abnormalities observed (pink) and those without abnormalities observed (blue) via ultrasound. Purple color denotes regions of overlap. **(B)** Cumulative mortality curves for embryos via ultrasound with and without abnormalities. Grey box represents the 2-week period where mortality increased at the fastest rate. Note, data from the singular hatchling is omitted from this figure.

Of the 120 embryonated eggs tracked in this study, only one survived to hatching (0.83%) and it was manually assisted out of the egg case after 205 days of incubation, ~45 days after its predicted hatching date (45). For embryos without an observed abnormality, lifespan ranged from 28 to 91 days (median 42 days) compared to 34 to 142 days (median = 63 days) for embryos with an identifiable abnormality (Figure 6A). Lifespan was significantly shorter for embryos where no abnormality was observed during ultrasound monitoring compared to those with an identifiable morphological issue (Wilcoxon U-test, W = 572, *p* < 0.0001). Incidence of mortality was particularly high between 35 to 56 days post-oviposition for embryos where no abnormality was observed. Cumulative mortality increased from 20 to 91.4% in this 21-day period of incubation (Figure 6B). Cumulative mortality increased more gradually for embryos with an observed abnormality with 90% mortality reached at 105 days post-oviposition. No abnormalities were detected via ultrasonography for the lone embryo that survived to hatching.

Flocculent material in the perivitelline fluid had a mean onset time of 45 ± 13 days post-oviposition and was also indicative of poor prognosis. In eggs where flocculent material manifested (*n* = 41, 34%), the result was 100% embryo mortality within 13 ± 16 days of its first observation. In half (50%) of these eggs, the embryos had one or more morphological abnormality identified (Figure 5B).

### 3.4 Genetic assignment

Of the 120 embryos, samples from 65 (54%) were sent for genetic testing to determine parentage. Of those, 17 had inconclusive results (i.e., amplification failed due to DNA degradation or too few markers amplified to conclusively determine parentage) and 48 were confirmed parthenotes, including the embryo that successfully hatched. At least one parthenote was produced by each of the four females. Parthenotes included embryos both with (*n* = 28) and without developmental deformities (*n* = 19).

## 4 Discussion

While its application in reproductive biology is widespread, in this study, ultrasonography was demonstrated to also be useful for monitoring embryo development and morbidity in an oviparous species. Ultrasonography allowed egg fertility to be assessed earlier in incubation and provided an alternative technique to monitor embryo growth and development compared to traditional methods to monitor embryonic development *in ovo*. Ultrasonography enabled a detailed view of the egg yolk, perivitelline fluid, and morphological features of the embryo, which yielded egg and embryo features that can be used as developmental thresholds of fertility and characteristics that were prognostic of morbidity when observed.

### 4.1 Ultrasonography as a tool to monitor embryonic development

Detection of early-stage embryos through ultrasonography was aided by conscientious egg handling and consistent egg orientation throughout the ultrasound exam procedure. Eggs were incubated and maintained in the same orientation when transferring between incubation and examination systems. This consistent gravitational force on the yolk preserves the animal-vegetal axis and maintains the animal pole and its germinal disc (or developing embryo) on the upper surface of the yolk mass. The predictable location for a developing embryo reduces the ultrasonography search space and facilitates discovery of small embryos.

Ultrasonography aided in earlier embryo identification when compared to a previous study where zebra shark eggs were candled weekly post-oviposition at the same facility’ [ultrasound: mean 30 ± 7 days; candling: 45 ± 12 days reported in Adams et al. (38)]. Most eggs in the present study were incubated for 3 to 4 weeks before the first fertility check, but through examination of a subset of eggs, it was demonstrated that embryos could be identified as early as 8 days post-oviposition. The longest time from oviposition to identification of an embryo was 47 days and occurred during the Covid-19 pandemic when schedule disruptions delayed regular ultrasound screening. Using 47 days as the upper threshold for determining egg fertility, facilities incubating zebra shark eggs at ~25°C should consider reserving resources after 7 week’s time. If no embryo has been identified by that point, it is likely that the egg is infertile, or suffered early embryonic death.

Ultrasonography was useful in monitoring embryonic growth. However, there were several limitations that prevent using the recorded measurements to calculate accurate growth rates. Capturing a true straight-line measurement of embryo length accurately and consistently was challenged by their nearly constant, active movements. For example, there were several instances where embryos decreased in length between time periods (i.e., they were measured “shorter” at a subsequent exam taken 1 week later), which highlights the limitations of this methodology. In addition, once embryos grew to a certain length (~8 cm), they were larger than the field of view of the ultrasound, preventing an accurate total length measurement. As an alternative, growth of embryos larger than ~8 cm could be monitored using head diameter (e.g., maximum distance between the eyes) or other features that can be more easily seen/captured in the frame of the ultrasound transducer (e.g., mouth gape). However, from a monitoring perspective, gross changes in growth from week to week were observable via ultrasonography.

In this study, ultrasonography was used to confirm fertility and identify egg and embryo characteristics that predicted morbidity when observed. However, there are other applications of this tool that could aid future studies on oviparous elasmobranch early life history and development in a non-invasive manner. Although not a focus, this study demonstrated how ultrasonography could be used to track heart rate and monitor changes in external gill filaments. Maternal provisioning could be inferred through ultrasonic measurements of the yolk mass as well as quantifying embryo yolk consumption across development. In a viviparous, lecithotrophic snake (*Vipera aspis*), ultrasound revealed development-related changes in embryonic volume that were largely attributed to mothers’ ability to meet embryonic hydration demands (46). Quantifying maternal-fetal relationships through use of non-invasive tools has implications for predicting how species may respond to climate change and/or resource limitation. Other applications of ultrasonography include quantifying aspects of embryo behavior during development. For example, ultrasonography revealed that late-term copperhead snakes (*Agkistrodon contortrix*) began performing caudal luring behaviors before birth (47), while embryos of the tawny nurse shark (*Nebrius ferrugineus*) were found to swim between the left and right uterus (48). Although these behaviors were documented in viviparous species, it demonstrates the utility that ultrasonography provides to characterize multiple aspects of the earliest stages of life, which can be applied to oviparous elasmobranch species as well.

### 4.2 Ultrasonography as a screening tool

Ultrasonography allowed for early identification of morphological abnormalities. These abnormalities were visually confirmed via necropsy in a subset of eggs to validate the ultrasound observations. Tail deformities were the most common abnormality and their prevalence among embryos, all which were confirmed parthenogenetic, suggests that this feature may be used as a biomarker of parthenogenesis and an early warning sign of embryonic mortality when observed. Development of this tool could have applications for conservation projects aiming to maintain genetically diverse populations of this endangered species. By having a method to screen for parthenotes at an early age, ultrasonography can enable resources to be directed towards embryos with greater chances of being heterozygotes resulting from sexual reproduction. However, absence of an observed abnormality on ultrasound does not preclude embryos from being unisexually produced, as demonstrated through the successful hatching of a parthenote in this study with no abnormalities noted on ultrasound.

Other common abnormalities, such as vesicles at the base of the yolk stalk, were equally prognostic of mortality. It is hypothesized that the vesicles visible as one or more anechoic foci during ultrasound exams are the result of focal delamination of the outer ectoderm from the inner enveloping layers of the yolk. Fluid accumulates in the foci and the pressure on the mesoderm, which include blood vessels, eventually restricts blood flow and cuts off circulation, which could account for the high mortality rate soon after a vesicle(s) was first observed.

A third commonly observed morbidity hallmark was the development of particulate material in the perivitelline fluid surrounding the embryo in the egg case. Embryos develop a hatching gland during early incubation that functions to effect eclosion through liquifaction of the semi-solid matrix that initially supports and surrounds the fragile yolk (31). As a result of this matrix breakdown, seawater can enter through the respiratory fissures and circulates through the egg case during the latter half of development (31). Eclosion is developmentally synchronized with the increase in size of the rapidly growing embryo and is proposed necessary to meet its increased oxygen demands. It is hypothesized that this particulate material is a result of incomplete breakdown of the semisolid matrix, due to improper or lack of development of the hatching gland. The particulate material makes the perivitelline fluid appear heterogeneous with small hyperechoic foci resembling “snow” in a snow globe when ultrasounding the egg. Failure to fully liquify the supporting internal matrix jelly would prevent eclosion and, in turn, could interfere with the ability of embryos to receive adequate oxygenation and likely lead to asphyxiation (49). Thus, like the other indicators of morbidity, an egg with streaming heterogeneous perivitelline fluid with particulate material on ultrasonography was confirmed at dissection and indicative of mortality.

While initially counterintuitive, embryos with no observed morphological abnormalities had shorter lifespans than embryos with an observed morphological abnormality. However, the median time to detect an abnormality (43 days post-oviposition) coincided with the median lifespan of embryos without a detectable morphological abnormality (42 days). Therefore, it is likely that many of these embryos perished before a morphological abnormality could be detected because of their small size. In particular, the two-week window around this time period (i.e., 43 ± 7 days) had the highest rates of mortality observed, suggesting that there is a key developmental checkpoint during this time frame that embryos must overcome for development to proceed. If an embryo is not able to overcome this barrier, embryonic death and autolysis proceeds relatively quickly with eggs fouling in less than 1 week. This key time period also aligns with a previous study in this same population of zebra sharks that reported a high degree of egg fouling (i.e., broken yolks) for the first 45 days post-oviposition (38). During that window, fertility was assigned as “unknown” since embryos could not be reliably observed via candling with the naked eye until day 45 post-oviposition. By contrast, eggs that were confirmed infertile maintained their yolk integrity for many weeks after oviposition (38). In light of the new information produced by this study, it is proposed that the broken yolks in Adams et al. (38) were the result of early embryo mortality. To support this hypothesis, a few eggs with broken yolks were examined outside the time frame of this study to determine if any signs of early embryonic development could be identified. Upon close examination embryos 1-3 mm total length were observed suggesting zebra shark eggs with broken yolks after 2–6 weeks of incubation may represent embryonic mortality rather than infertility (Supplementary File 12).

While all embryos with an observed abnormality died, the absence of an abnormality after 42 days post-oviposition did not guarantee long-term viability or sexual reproduction as demonstrated by the successful development and hatching of one parthenogenetically-produced offspring. This embryo developed completely and the egg and embryo morbidity hallmarks described above were not observed; however, incubation was protracted and it was manually extracted from the egg case to prevent hatching-associated mortality. Due to the lack of heterozygous offspring in this study, the timeline and progression of normal development was not available for direct comparison. Studies on zebra shark growth post-hatch have shown that parthenogenetic offspring do not grow equally (both in length and mass) as their heterozygous counterparts (45). Although no clear differences in hatchling morphometrics were reported, it is possible that parthenogenetic embryos exhibit the same delayed growth rate during embryonic development as was demonstrated for homozygous hatchlings. Understanding if parthenotes develop at a slower rate *in ovo* would allow for embryo size and yolk utilization thresholds to be used as biomarkers of parthenogenesis and could reduce the need for genetic confirmation. Access to sexually-developing, heterozygous embryos is needed to describe normal stages of embryonic development and their developmental timeline for comparison to parthenogenetically-developing embryos incubated at the same temperature.

Parthenogenesis, as a phenomenon, has generally been studied in aquariums by accident and through singular events because of unexpected births when females are kept in single-sex populations. Although not an original goal of the study, the data collected demonstrate that the probability that a parthenote successfully develops to hatching is extremely small (0.83%) and is comparable to parthenogenesis hatch rates in another oviparous species, the whitespotted bamboo shark *Chiloscyllium plagiosum* (0.71%) (50). This rate for zebra sharks is calculated with the assumption that all untested embryos in the study were parthenotes. Although small, this is likely an overestimation as our dataset included only the eggs where embryos were large enough to be identified using ultrasonography (i.e., not all eggs of all females were included in this dataset). This low hatching rate aligns with the seemingly low reports of parthenote births observed across taxa, that often occur as only single observations per species (51–53). Despite the low probability of successful development to hatch, many of the observed embryos were confirmed parthenotes, indicating this mode of reproduction occurs more frequently than previously thought in the zebra shark. A previous study with a singleton female zebra shark documented a parthenogenesis hatch rate higher than our study (~12%) (54). Due to the high mortality rate for parthenotes (especially in early development), future studies should assess all eggs during this period to more accurately determine the prevalence of this phenomenon.

## Data availability statement

The raw data supporting the conclusions of this article will be made available by the authors, without undue reservation.

## Ethics statement

The requirement of ethical approval was waived by Aquarium of the Pacific Research Advisory Committee for the studies involving animals because study data was obtained from routine care. The studies were conducted in accordance with the local legislation and institutional requirements. Written informed consent was obtained from the owners for the participation of their animals in this study.

## Author contributions

LA: Conceptualization, Investigation, Methodology, Project administration, Supervision, Writing – review & editing. JW: Conceptualization, Investigation, Methodology, Project administration, Supervision, Writing – original draft, Writing – review & editing. BG: Data curation, Investigation, Project administration, Visualization, Writing – review & editing. RM: Data curation, Investigation, Project administration, Visualization, Writing – review & editing. LL: Data curation, Methodology, Visualization, Writing – review & editing. KF: Data curation, Formal analysis, Resources, Validation, Writing – review & editing. KL: Conceptualization, Data curation, Formal analysis, Investigation, Methodology, Project administration, Writing – original draft, Writing – review & editing.

## Footnotes

^1^http://www.geneious.com
