# Supplementary material for: Monitoring egg fertility, embryonic morbidity, and mortality in an oviparous elasmobranch using ultrasonography
Source: Front Vet Sci. 2024 Jul 30;11:1410377. doi: 10.3389/fvets.2024.1410377 (PMC11319157; doi:10.3389/fvets.2024.1410377)
Supplement: Supplementary file 1 [file Data_Sheet_1.zip › Datasheet 1/Supplementary Material Legends.PDF]

Supplemental File Legends

Supplemental Material 1. Zebra shark (*Stegostoma tigrinum*) egg tagging method is shown using a conventional retail tagging gun. A) The laminated number is pierced prior to application on the egg. B) The tagging gun is then inserted through the non-hatching end of the egg case. C) example of egg case with identification number attached.

Supplemental Material 2. Zebra shark (*Stegostoma tigrinum*) egg case submerged in seawater in a Kritter Keeper at the examination room. Probe orientation on the top of the egg case is shown. Eggs were previously incubated in the same orientation to allow migration of the animal pole to the top side of the egg for easy viewing.

Supplemental Material 3. Zebra shark (*Stegostoma tigrinum*) eggs were evaluated for their yolk integrity and homogeneity of perivitelline fluid at each ultrasound examination. White arrow(s) denote particulate material in perivitelline fluid. A) Egg with intact yolk surrounded by perivitelline fluid that does not contain particulate material. B-D) Progressive loss of yolk integrity and increase in particulate material in perivitelline fluid.

Supplemental Material 4. Videos of various degrees of Zebra shark (*Stegostoma tigrinum*) embryo movement as seen on ultrasound. A) Steady movement was categorized as continuous movement by the embryo. B) Slow movements were categorized as embryos that displayed weak movements. C) Non-moving embryos were categorized as dead. Videos can be played by hovering the mouse over each panel.

Supplemental Material 5. Example video of small zebra shark (*Stegostoma tigrinum*) embryo (0.35 cm) first observed at 24 days post-oviposition. Video can be played by hovering the mouse the graphic.

Supplemental Material 6. Videos documenting absence (A) and presence (B-C) of particulate material as viewed on ultrasound compared to necropsy for zebra shark (*Stegostoma tigrinum*) eggs (D). Extent of flocculent material increases from panel B to C on ultrasound. Videos can be played by hovering the mouse over each panel.

Supplemental Material 7. Examples of growth progression and measurements for three zebra shark (*Stegostoma tigrinum*) embryos (top: Egg #308, A-C; middle: Egg #509, D-F; bottom: Egg #524, G-I) across three time points (left to right).

Supplemental Material 8. Video recordings for the hatched zebra shark (*Stegostoma tigrinum*) embryo (Egg 207) documenting *in ovo* heart beat (A) and buccal pumping (B). Videos can be played by hovering the mouse over each panel.

Supplemental Material 9. Videos of zebra shark (*Stegostoma tigrinum*) embryos with tail deformities ranging from kinked or "clubbed" at the end (A, C) to embryos with a visible "bend" (B, D). Embryos depicted ranged in age from 49 (A), 60 (B) and 65 (C,D) post-oviposition. Note that anechoic bubbles were also concomitantly observed on ultrasound. Videos can be played by hovering the mouse over each panel.

Supplemental Material 10. Bubble appearance and enlargement from time 1 (left-side panels; A, C) to time 2 (right side panels; B, D) as seen on ultrasound for two different zebra shark (*Stegostoma tigrinum*) eggs (Egg # 497 #527).

Supplemental Material 11. Bubble diameter over successive exams denoted by white arrows. Different colored lines represent unique zebra shark (*Stegostoma tigrinum*) eggs.

Supplemental Figure 12. Zebra shark (*Stegostoma tigrinum*) egg #396 was laid on June 9th, 2022. On June 28th (19 days post-oviposition) the egg underwent its first ultrasound examination and no embryo was found. One week later on July 5th (26 days post-oviposition), the yolk was found to be degraded and the egg case had a slimy exterior indicating the egg had fouled. The egg was wrapped in moist paper towels and shipped on ice packs for necropsy at the University of Delaware as part of another study. Upon dissection, a 1-2 mm embryo was found (indicated by white arrow) confirming the egg was indeed fertile, despite no embryo being seen on ultrasound at this early age.
