# Supplementary material for: Monitoring egg fertility, embryonic morbidity, and mortality in an oviparous elasmobranch using ultrasonography
Source: Front Vet Sci. 2024 Jul 30;11:1410377. doi: 10.3389/fvets.2024.1410377 (PMC11319157; doi:10.3389/fvets.2024.1410377)
Supplement: Supplementary file 1 [file Data_Sheet_1.zip › Datasheet 1/Supplementary Tables.docx]

Supplemental Table 1. Characteristics of microsatellite loci developed for this study.

| Locus | Primer sequence 5' – 3' | Repeat motif | A | Size range (bp) | GenBank Accession # |
| --- | --- | --- | --- | --- | --- |
| Sfa325 | F: CTGTTTTGGCCTCTGCTGAT | (TG)_20_ | 18 | 216-288 | OR509286 |
|  | R: TTCCCTGCCTTAGAGCAAGA |  |  |  |  |
| Sfa371 | F: TGATCGGTGCAAGTGAATGT | (ATC)_16_ | 10 | 237-267 | OR509287 |
|  | R: CGAGCCAATGAATTACTCCTG |  |  |  |  |

A = no. of alleles. Forward primers were 5’-tailed with ­­­­­­­­­TGTAAAACGACGGCCAGT, while reverse primers were 5’ tailed with GTGTCTT.

Supplemental Table 2. Time to first observance of significant developmental landmarks via ultrasound for incubating zebra shark (*Stegostoma tigrinum*) embryos.

| Feature | Mean ± SD | Earliest 1^st^ Observance | Latest 1^st^ Observance |
| --- | --- | --- | --- |
| External Gill Filaments | 66 ± 19 days | 34 days | 100 days |
| Heart Beat | 87.5 days | 61 days | 114 days |
| Mouth | 108.5 ± 8 days | 103 days | 114 days |
