# Supplementary figures and images for: Monitoring egg fertility, embryonic morbidity, and mortality in an oviparous elasmobranch using ultrasonography
Source: Front Vet Sci. 2024 Jul 30;11:1410377. doi: 10.3389/fvets.2024.1410377 (PMC11319157; doi:10.3389/fvets.2024.1410377)

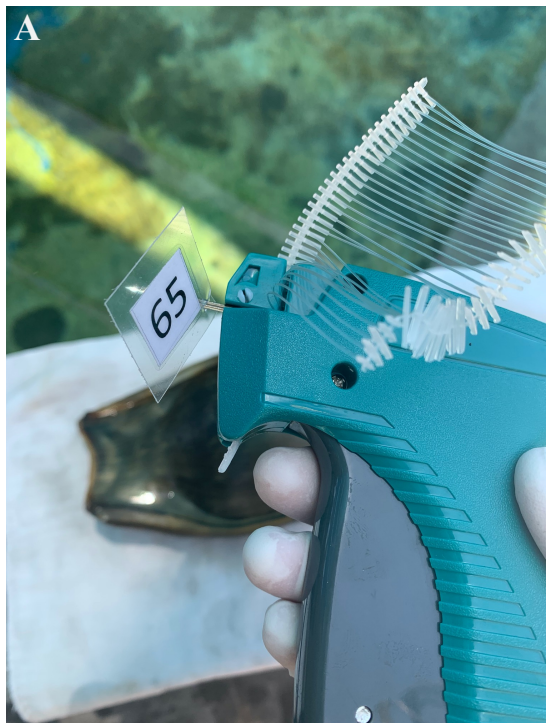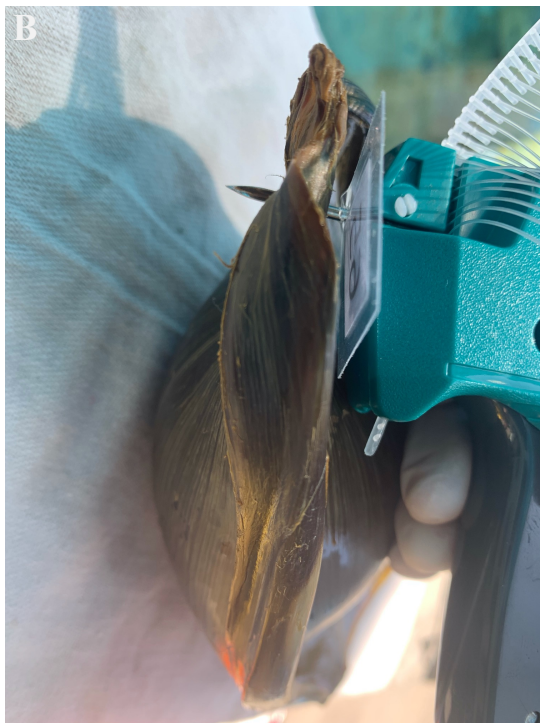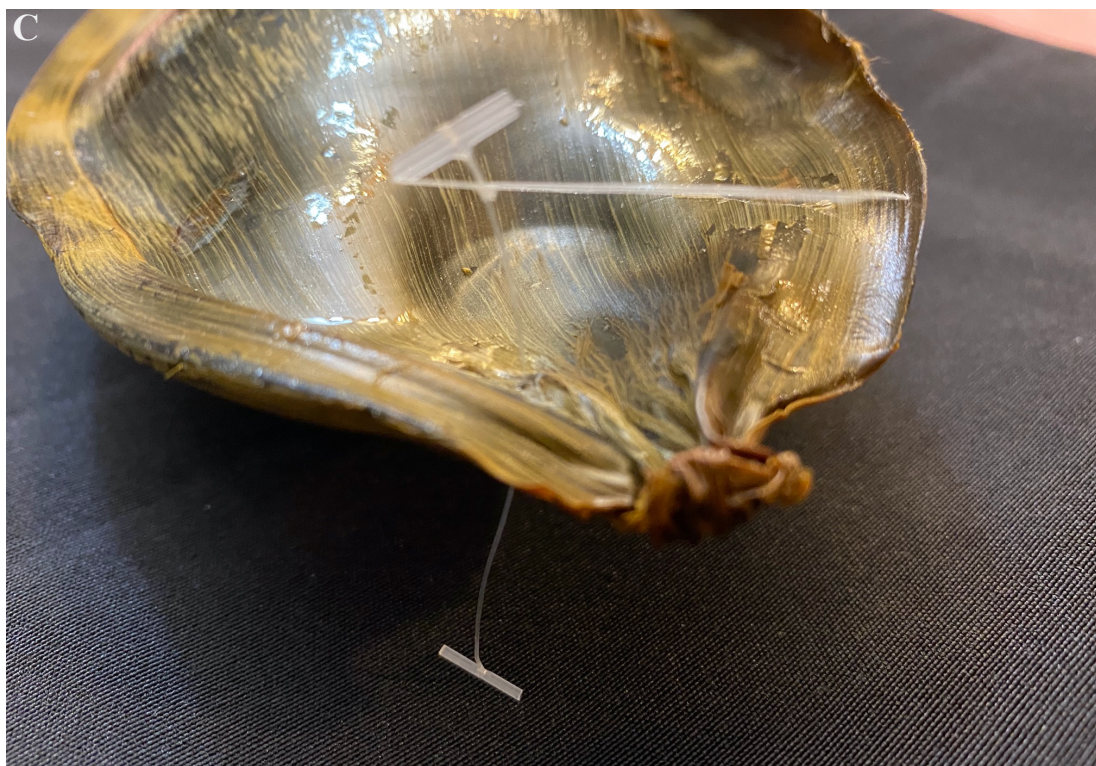

Supplement: Supplementary file 1 [file Data_Sheet_1.zip › Datasheet 1/Supplementary Material 1.PDF]

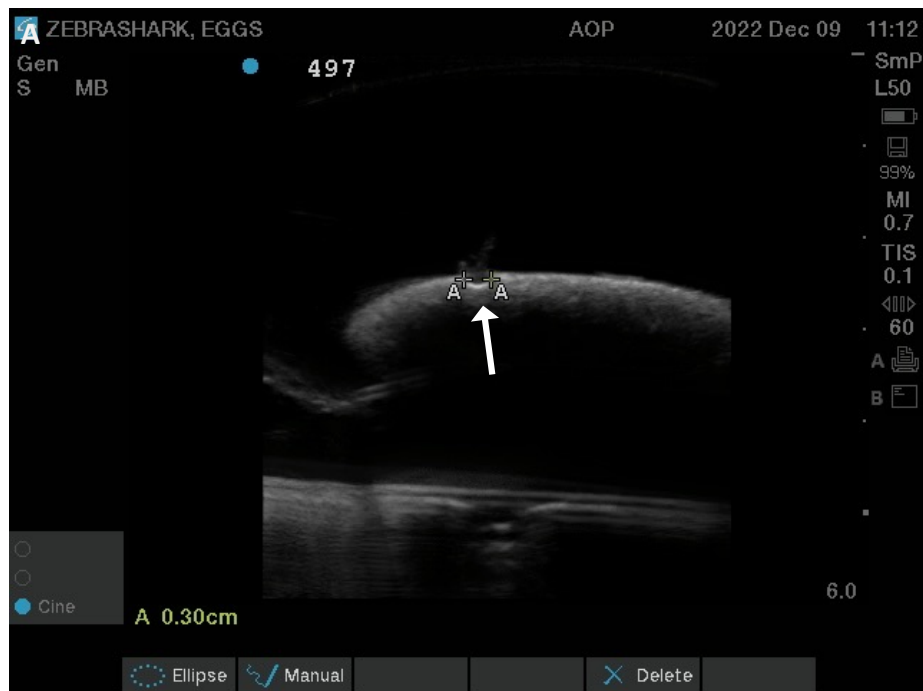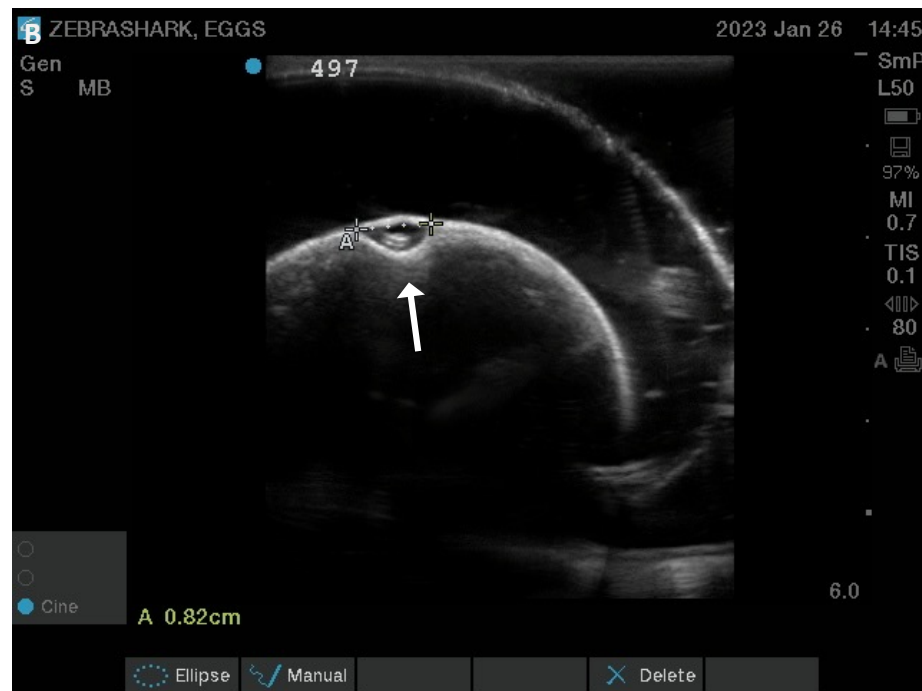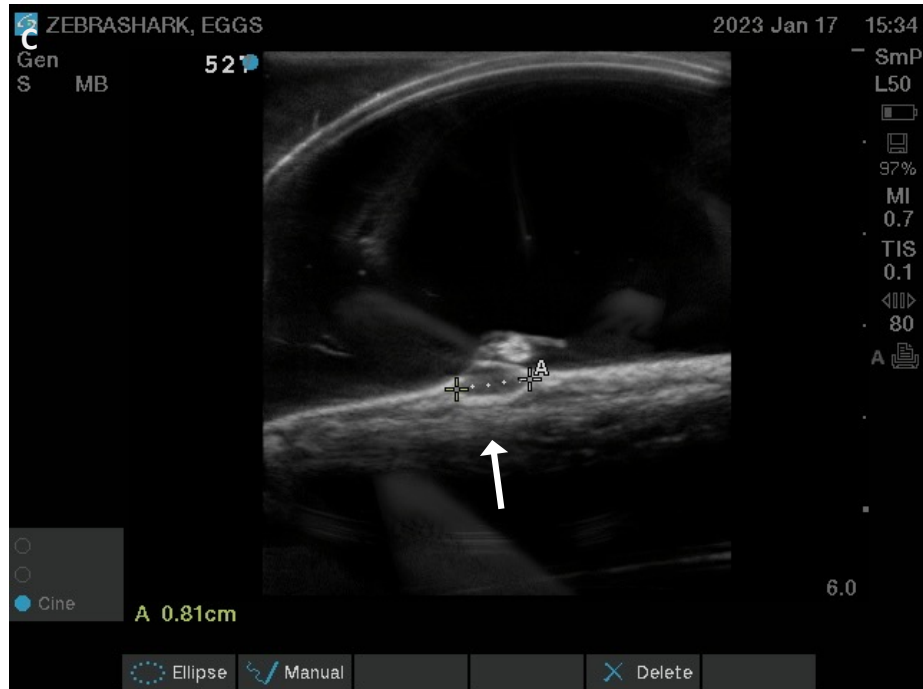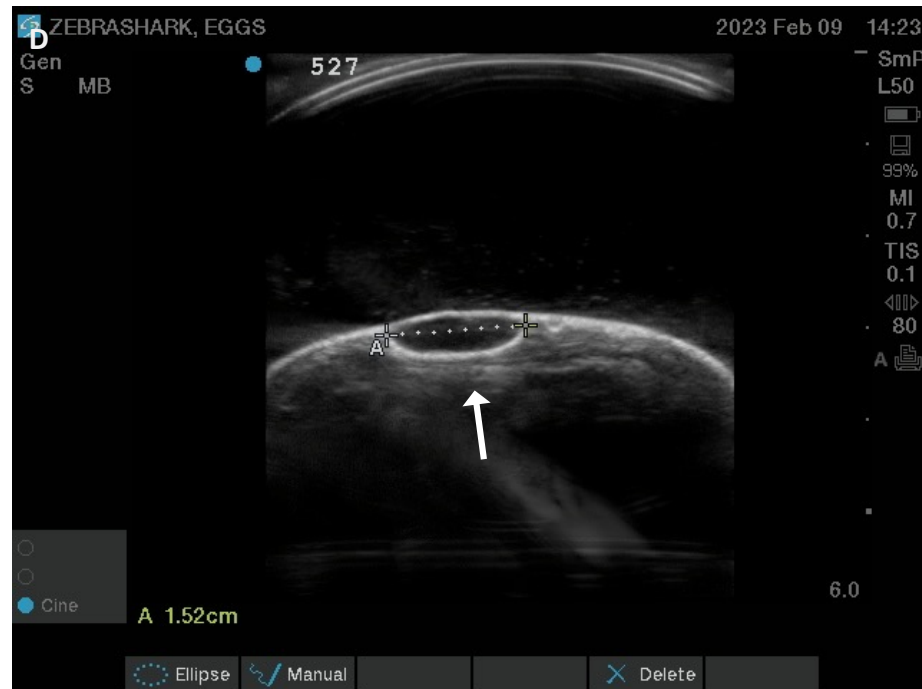

Supplement: Supplementary file 1 [file Data_Sheet_1.zip › Datasheet 1/Supplementary Material 10.PDF]

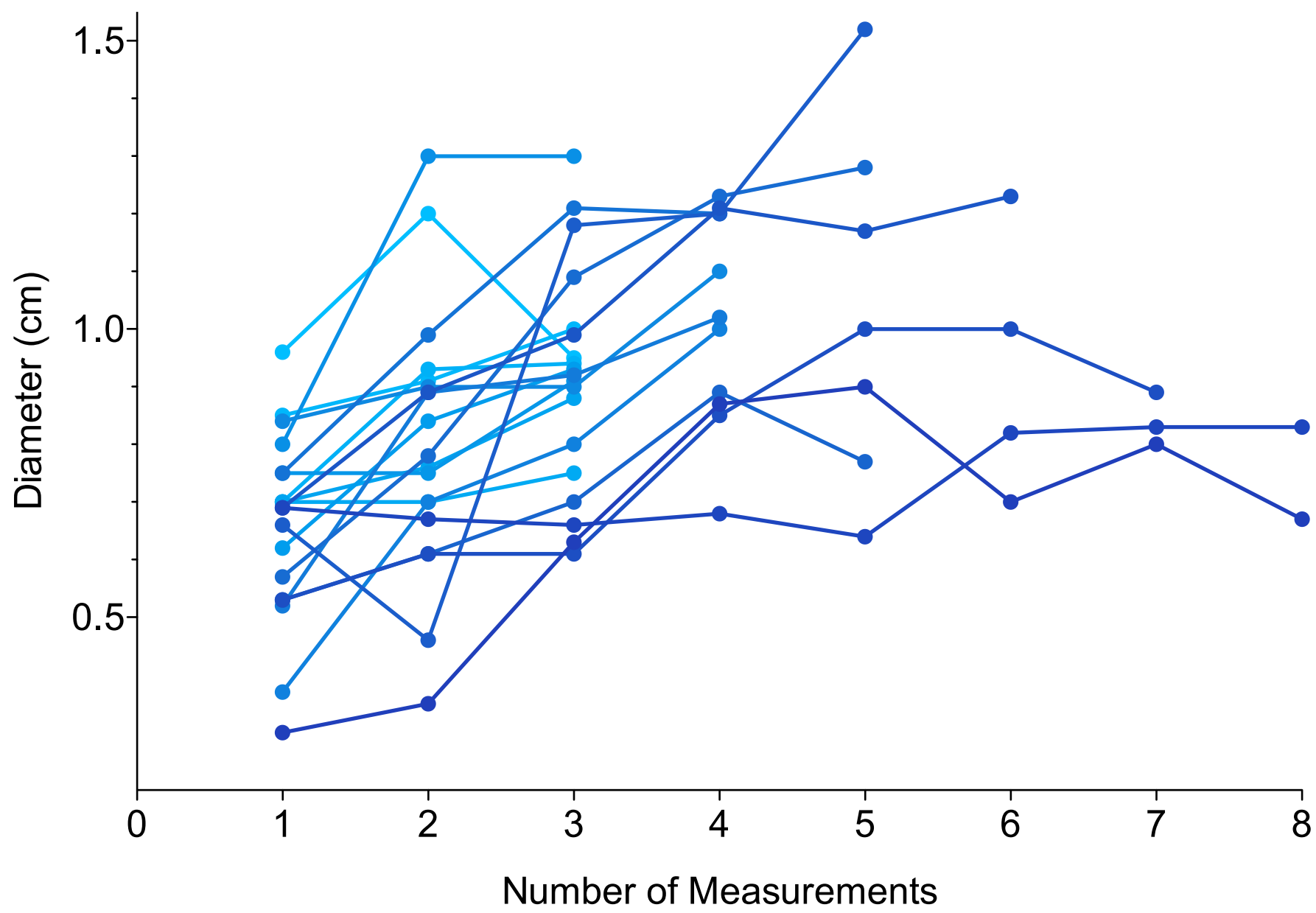

Supplement: Supplementary file 1 [file Data_Sheet_1.zip › Datasheet 1/Supplementary Material 11.PDF]

Egg # 396

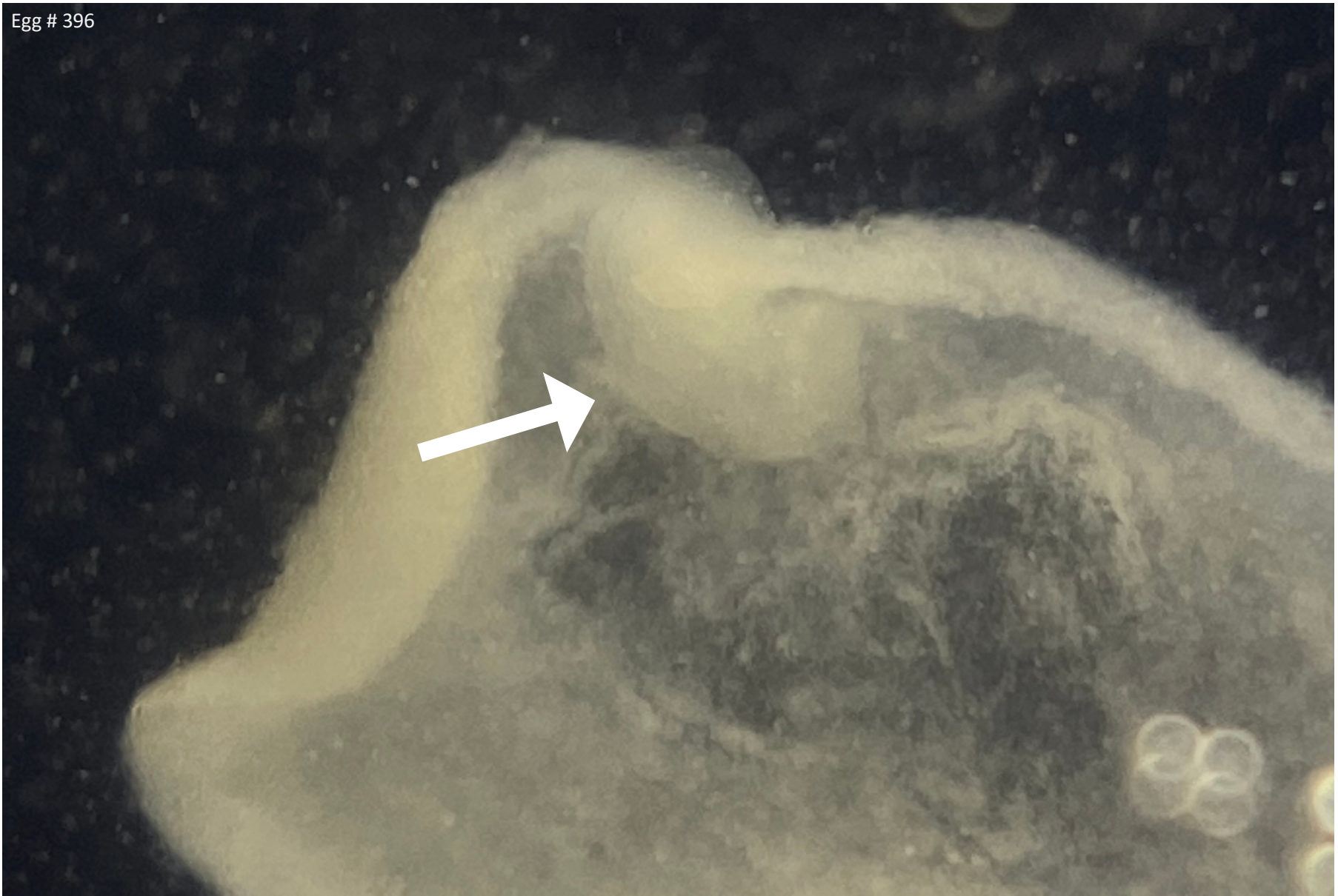

Supplement: Supplementary file 1 [file Data_Sheet_1.zip › Datasheet 1/Supplementary Material 12.PDF]

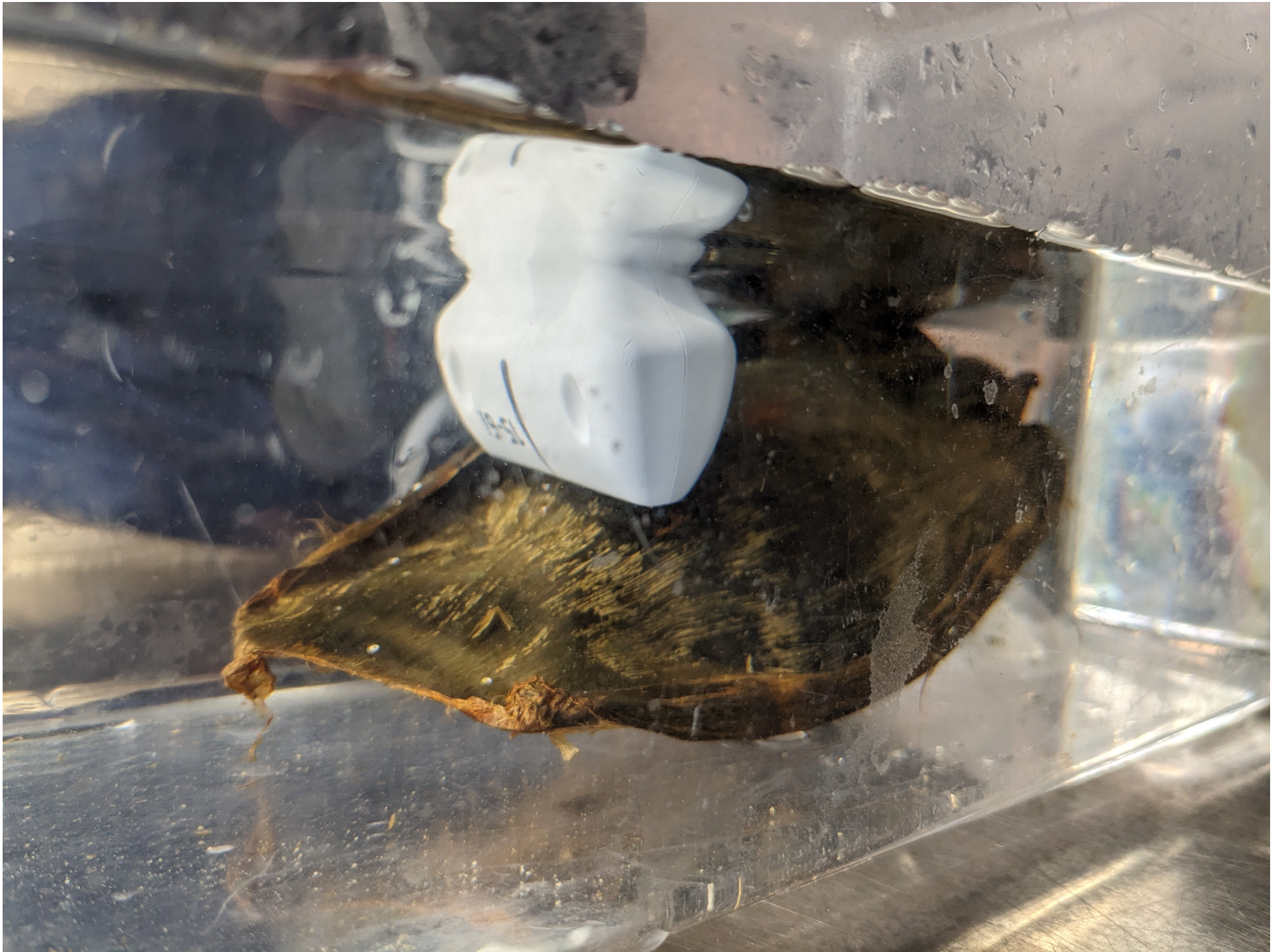

Supplement: Supplementary file 1 [file Data_Sheet_1.zip › Datasheet 1/Supplementary Material 2.PDF]

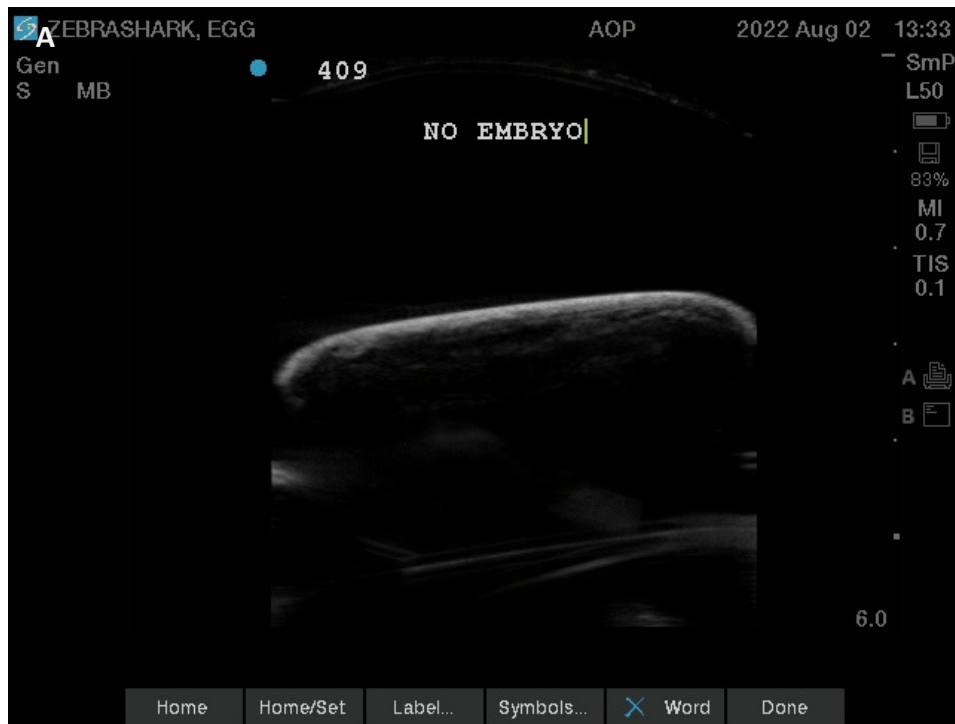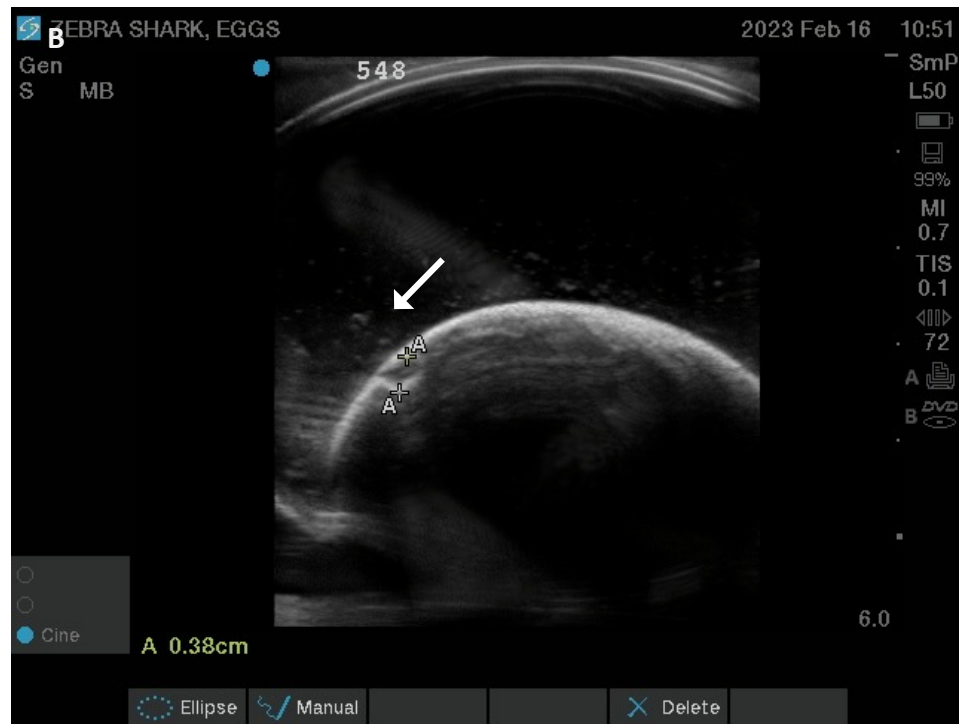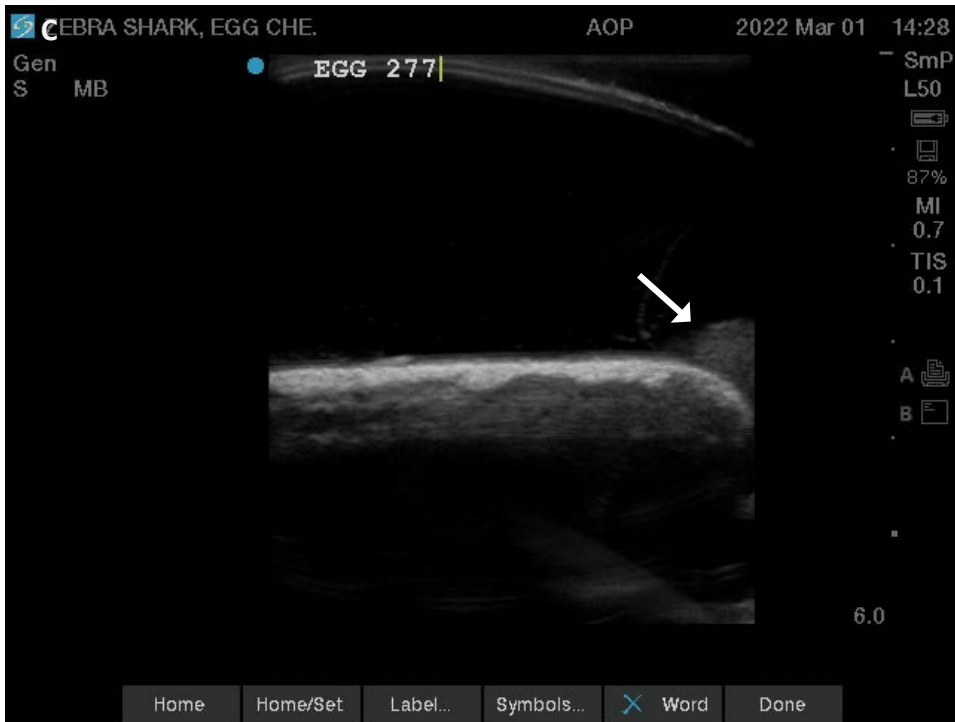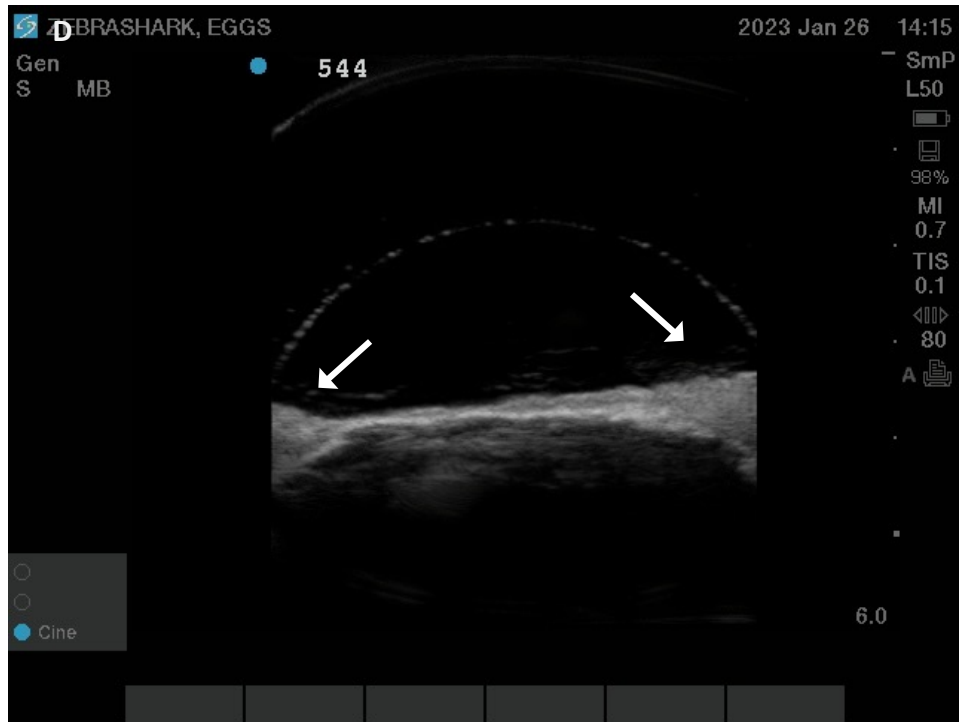

Supplement: Supplementary file 1 [file Data_Sheet_1.zip › Datasheet 1/Supplementary Material 3.PDF]

## Slide 1
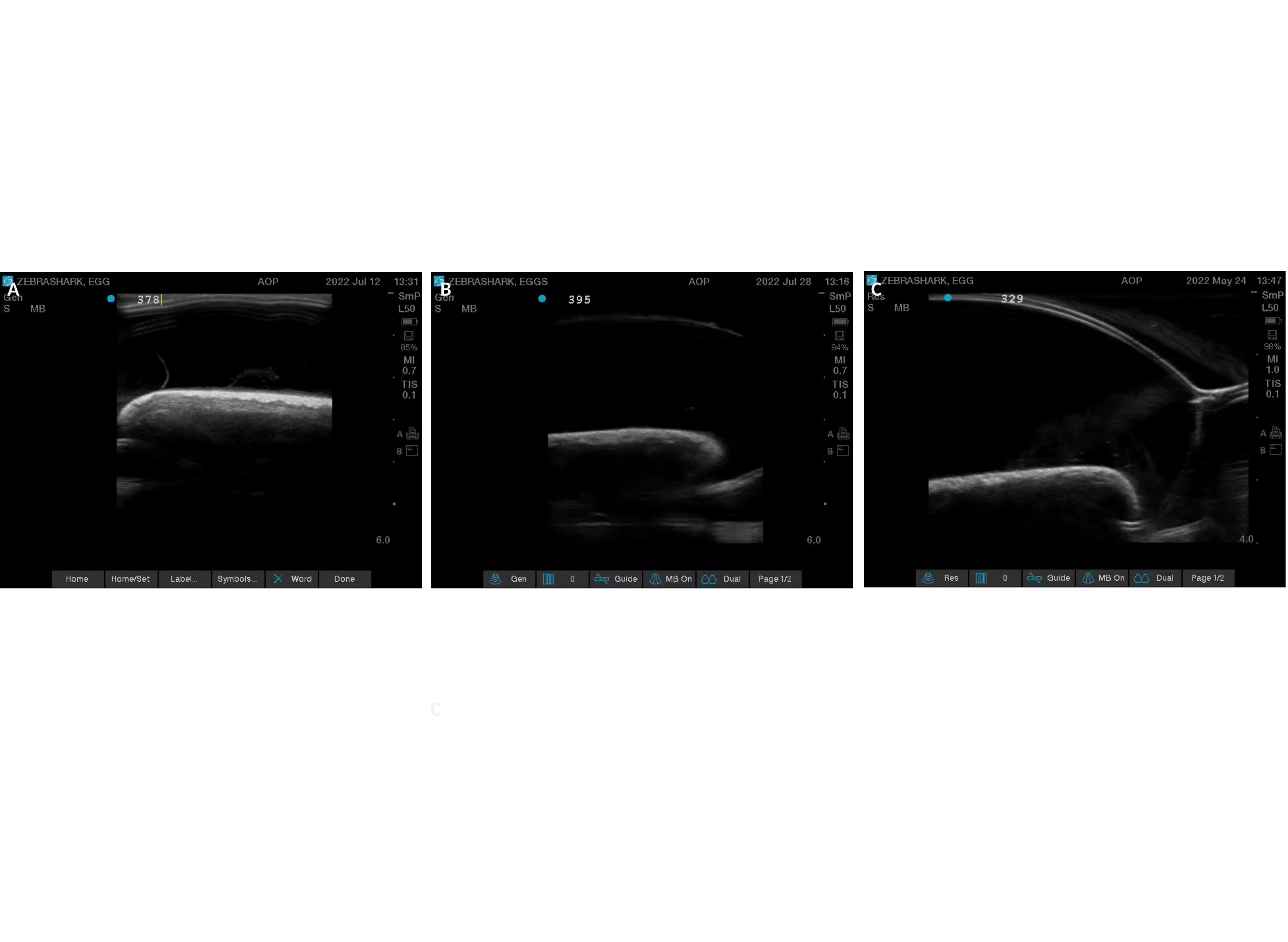

A
C
A
B
C

Supplement: Supplementary file 1 [file Data_Sheet_1.zip › Datasheet 1/Supplementary Material 4.pptx]

## Slide 1
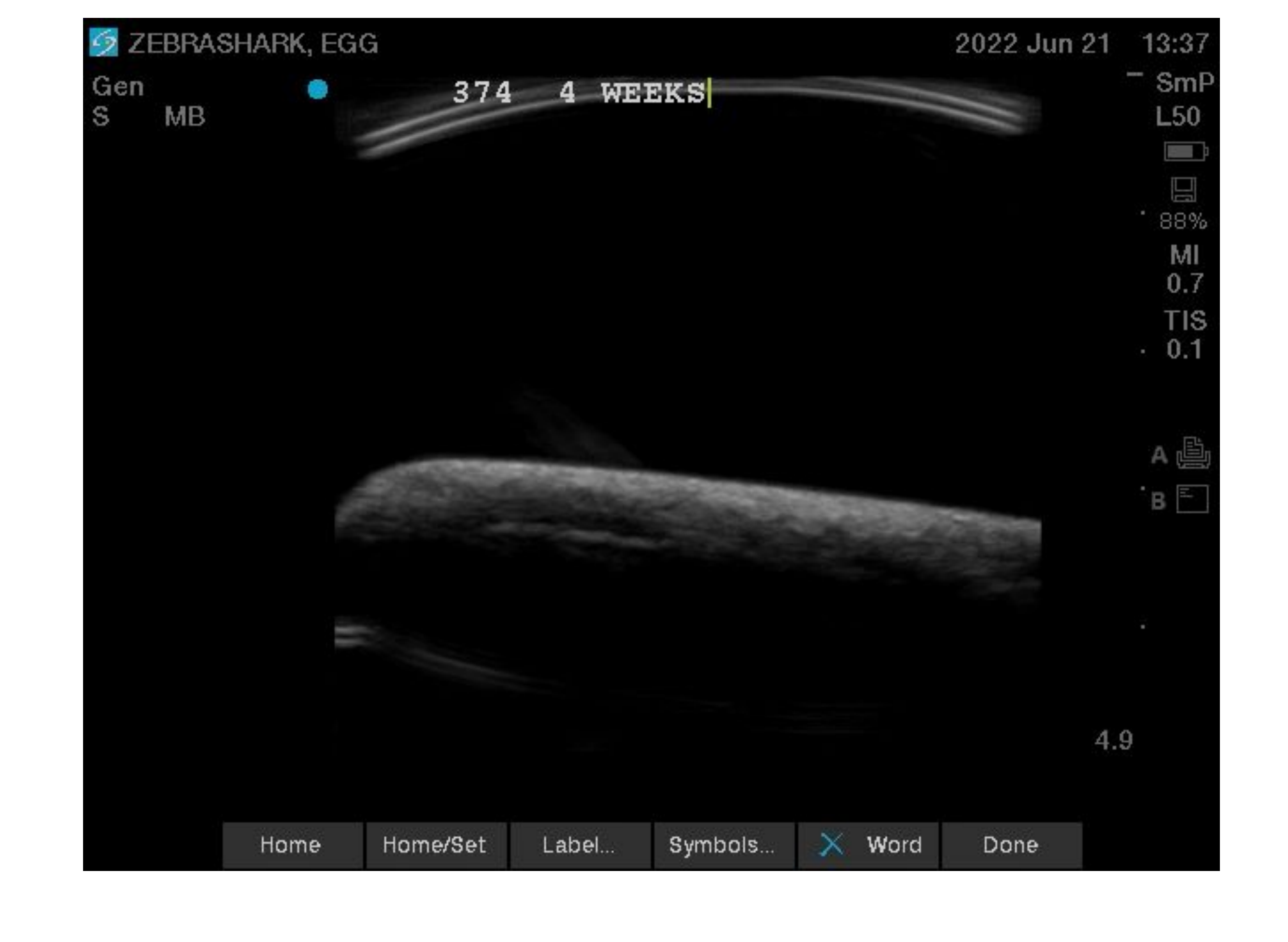

Supplement: Supplementary file 1 [file Data_Sheet_1.zip › Datasheet 1/Supplementary Material 5.pptx]

## Slide 1
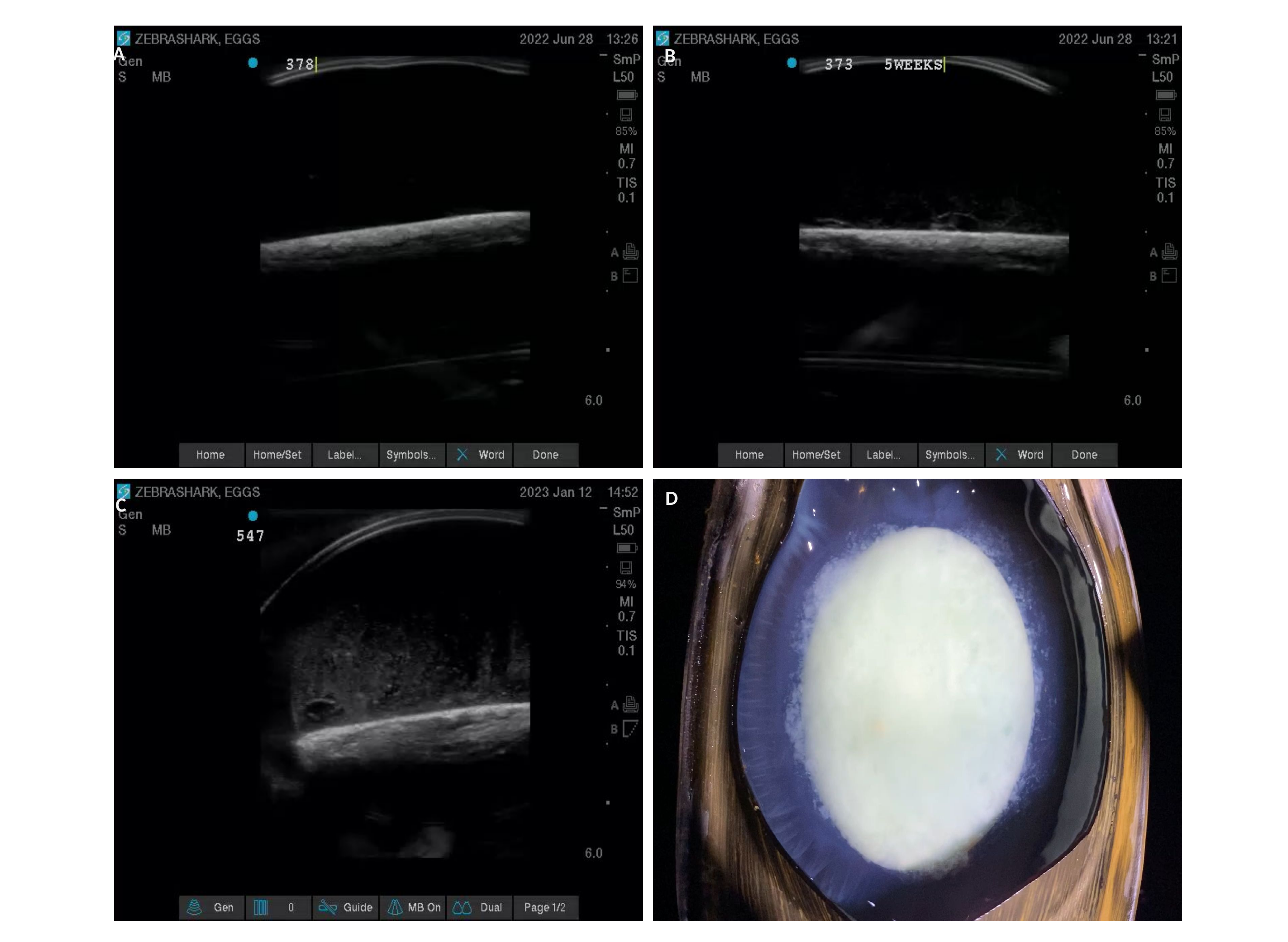

A
B
D
C
A
C

Supplement: Supplementary file 1 [file Data_Sheet_1.zip › Datasheet 1/Supplementary Material 6.pptx]

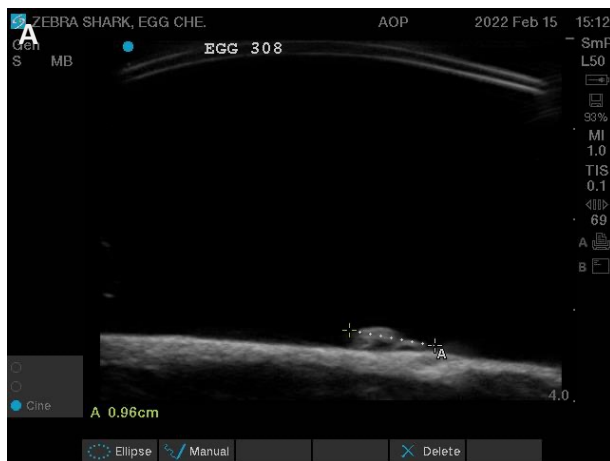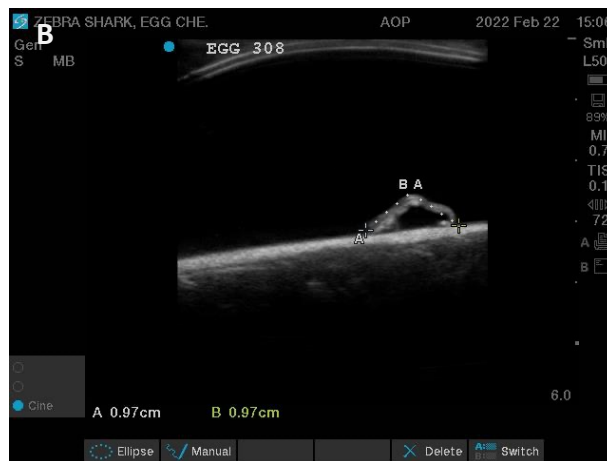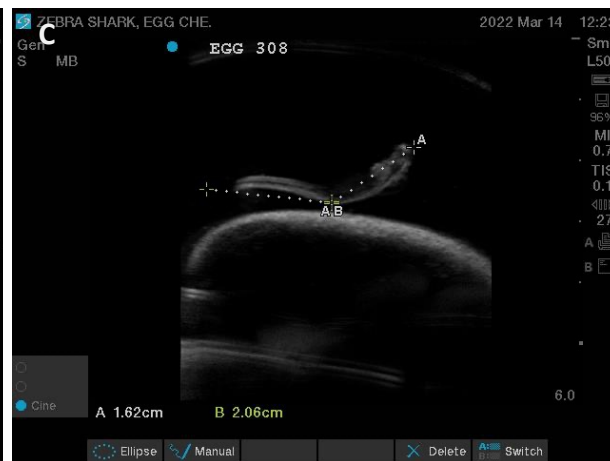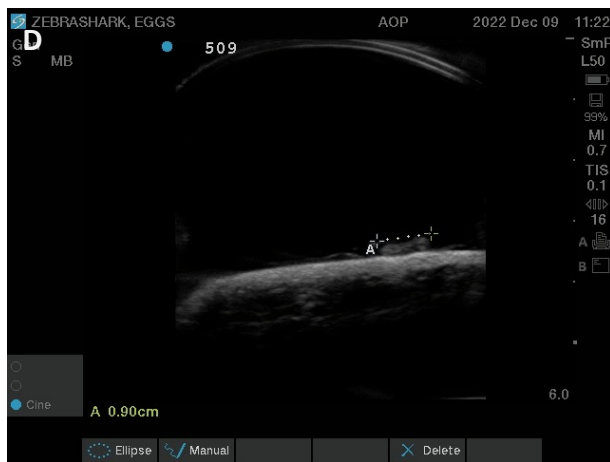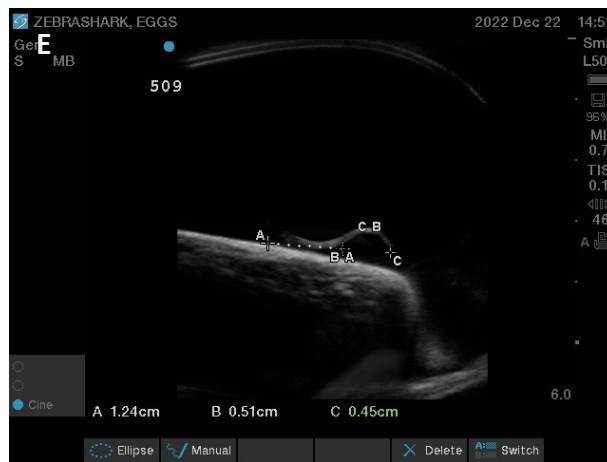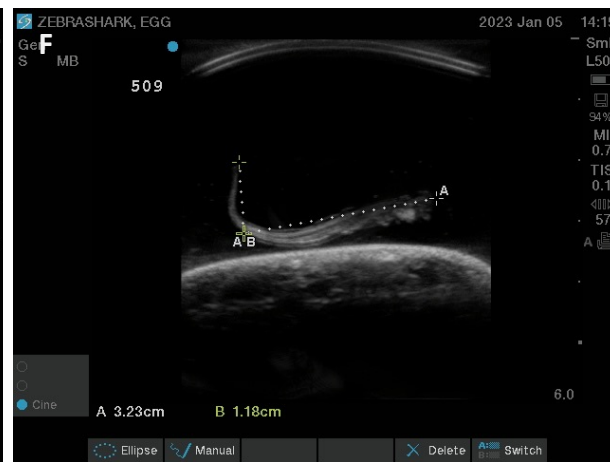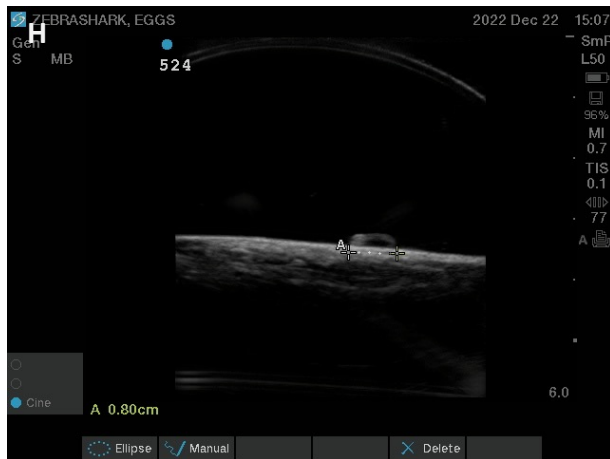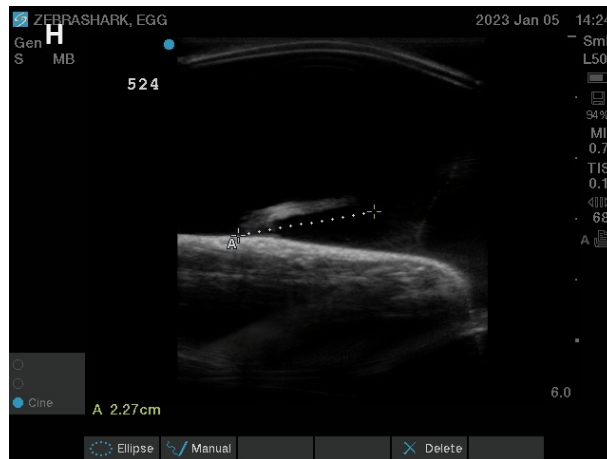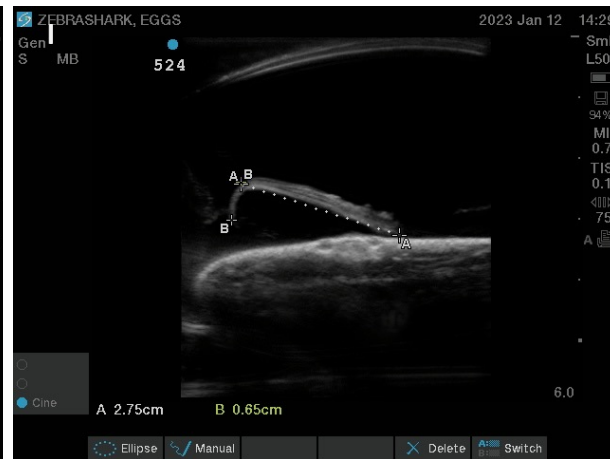

Supplement: Supplementary file 1 [file Data_Sheet_1.zip › Datasheet 1/Supplementary Material 7.PDF]

## Slide 1
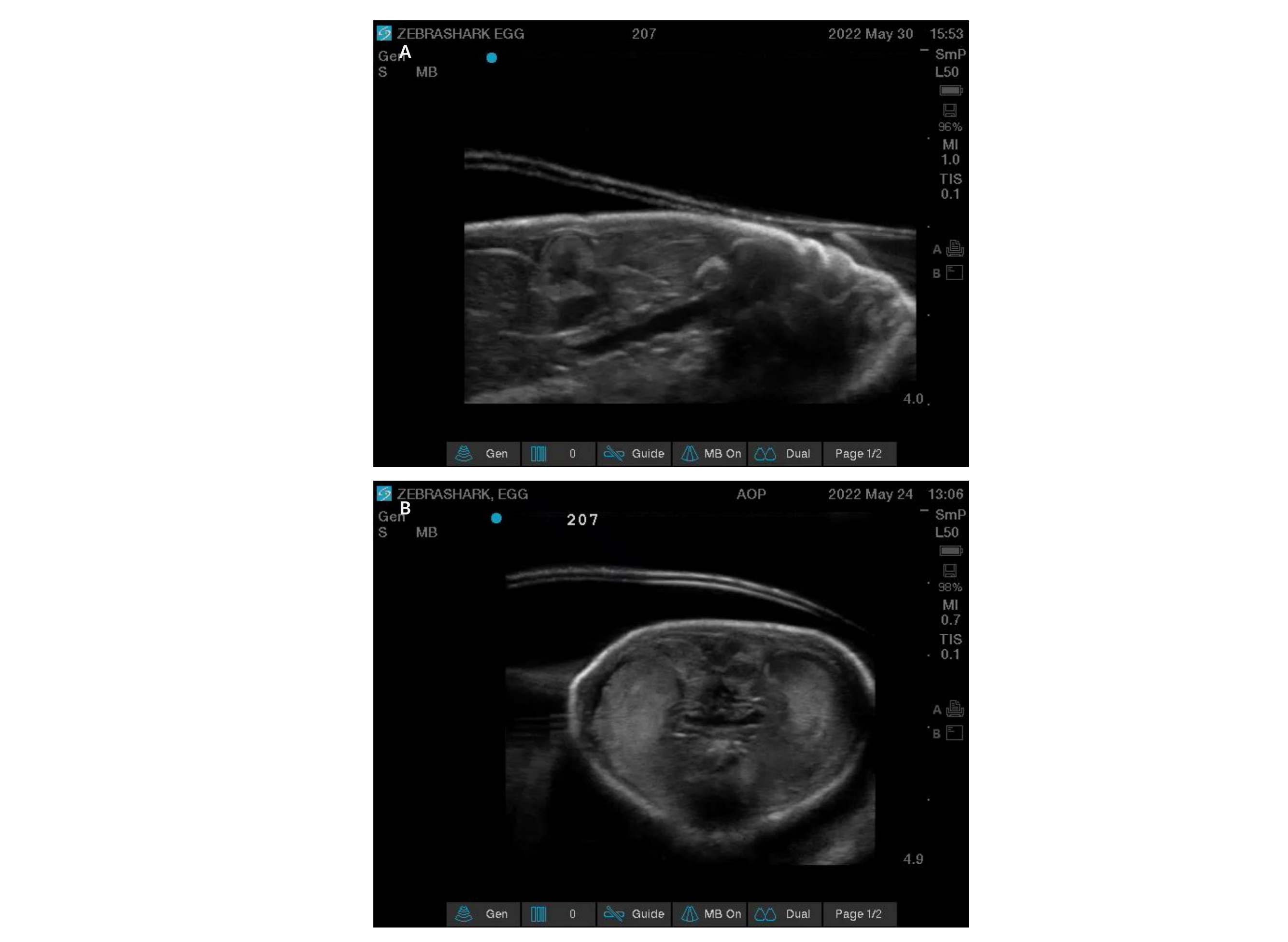

A
B

Supplement: Supplementary file 1 [file Data_Sheet_1.zip › Datasheet 1/Supplementary Material 8.pptx]

## Slide 1
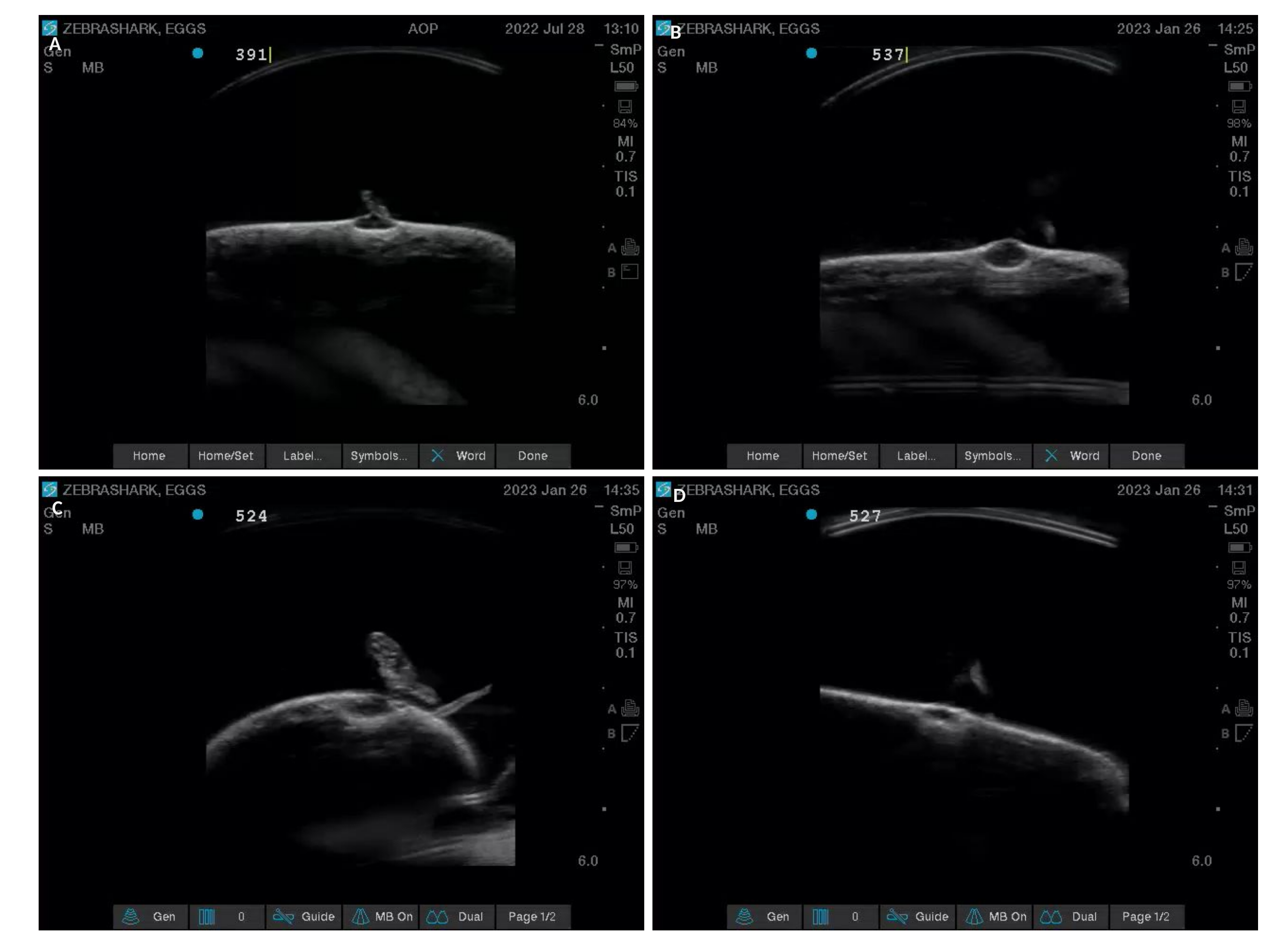

B
A
D
C
A
C

Supplement: Supplementary file 1 [file Data_Sheet_1.zip › Datasheet 1/Supplementary Material 9.pptx]
